# Design of Poly-Catechol Biodynamers for Potentiation of Antibiotic Efficacy against Drug-Resistant Bacteria

**DOI:** 10.1021/acs.biomac.5c02130

**Published:** 2026-02-23

**Authors:** Lena Zeroug-Metz, Kristela Shehu, Justine Bassil, Justin Podlecki, Philipp Sonntag, Marcus Koch, Anastasia Christoulaki, Eric Buhler, Anna K. H. Hirsch, Annette Kraegeloh, Marc Schneider, Sangeun Lee

**Affiliations:** 1 Pharmaceutical Materials and Processing, Department of Pharmacy, 9379 Saarland University, Campus C4.1, Saarbrücken 66123, Germany; 2 Biopharmaceutics and Pharmaceutical Technology, Department of Pharmacy, 9379 Saarland University, Campus C4.1, Saarbrücken 66123, Germany; 3 28391 INM − Leibniz Institute for New Materials, Campus D2.2, Saarbrücken 66123, Germany; 4 Helmholtz Institute of Pharmaceutical Research Saarland (HIPS) − Helmholtz Centre for Infection Research (HZI), Campus E8.1, Saarbrücken 66123, Germany; 5 Medicinal Chemistry, Department of Pharmacy, 9379 Saarland University, Campus E8.1, Saarbrücken 66123, Germany; 6 PharmaScienceHub (PSH), 9379 Saarland University, Saarbrücken 66123, Germany; 7 120190 University of Applied Sciences, htw saar, Goebenstr.40, Saarbrücken 66117, Germany; 8 Laboratoire Matière et Systèmes Complexes (MSC), UMR CNRS 7057, Physics Department, 555089 Université Paris Cité, Bâtiment Condorcet Paris 75013, France

## Abstract

Catechol-modified polymers, such as DOPA-functionalized systems, have recently gained significant interest for a variety of biomedical applications, particularly in their role as antibacterial adjuvants due to their oxidative activity and ability to generate reactive oxygen species (ROS). Current catechol-functionalized polymers, however, often suffer from a restricted number of catechol groups, limited biocompatibility and solubility, and low stability due to the rapid oxidation under physiological conditions. In this study, we developed a water-soluble, biocompatible DOPA-modified biodynamer (DOPA-BD), leveraging the principles of constitutional dynamic chemistry (CDC). DOPA-BD was synthesized via polycondensation of DOPA-hydrazide and the hexaethylene glycol-conjugated carbazole dialdehyde (CA-HG), forming dynamic imine and acylhydrazone linkages between the monomers. As a result of its dynamic covalent backbone, DOPA-BD exhibits biodegradability and undergoes pH-responsive degradation under mildly acidic conditions typically found at infection sites, leading to a more than 3-fold increase in DOPA-hydrazide release compared to physiological pH. Interestingly, driven by CDC, DOPA-BD folds into a nanorod structure with a hydrodynamic diameter of ∼7.8 nm, surrounded by HG chains that offer water solubility and biocompatibility. Moreover, the incorporation of the DOPA-derivative in each repeating unit yields a polymer with exceptionally high catechol content, which remains stable and resistant to oxidation for 72 h in physiological buffer conditions. Regarding its antibacterial applicability, DOPA-BD demonstrated synergistic antibacterial activity with Azithromycin (AZM) against AZM-resistant *E. coli*, enhancing the antibiotic’s efficacy by 4-fold. Our study indicates that DOPA-BD induces ROS production in the respective bacterial strain, suggesting ROS generation as one of the possible mechanisms contributing to the observed synergy. Overall, DOPA-BD represents a promising alternative strategy to potentiate antibacterial activity against resistant strains, holding strong potential for future antibacterial applications.

## Graphical abstract

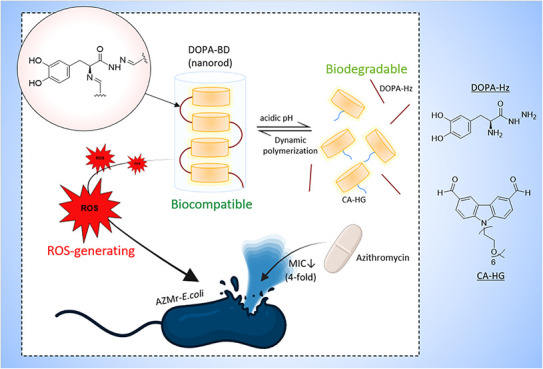

## 1 Introduction

Within recent years, catechol moieties have attracted significant attention in biomedical research due to their diverse chemical reactivities, such as π–π stacking, metal coordination, and covalent bonding, which facilitate a wide range of functions, including adhesion, self-healing, sensing, and drug delivery.^1–8^ In nature, l-3,4-dihydroxyphenylalanine (l-DOPA), a multifunctional catecholamine best known as a biosynthetic precursor to melanin, plays a critical role in the byssus of the blue mussel (*Mytilus edulis*), where DOPA-rich proteins form metal-coordinated cross-links with Fe­(II) and Fe­(III), resulting in durable adhesive structures.^9,10^ This ability has inspired a range of biomedical applications, including tissue adhesives, implant coatings, and wound-healing materials.^7,11–15^

In addition, naturally occurring catechol-containing compounds, including tannic acid, curcumin, and DOPA, are known to exert antimicrobial activity, which has led to growing interest in incorporating catechol units into advanced antibacterial platforms.^16–21^ Among the various functional properties of catechols, their oxidative conversion to *o*-quinones is particularly notable, as it leads to the generation of ROS such as hydrogen peroxide (H_2_O_2_) and hydroxyl radicals (•OH).^7,21–24^ These ROS exhibit well-documented cytotoxic effects, particularly in microbial systems, where they induce damage to proteins, lipids, and nucleic acids, ultimately resulting in bacterial cell death.^7,24^ In particular, integrating catechol moieties into polymeric systems has been shown to enhance their antibacterial potential by enabling localized and sustained ROS generation.^7,11,25^ The structural tunability and multivalency of catechol-functionalized polymers can allow precise control over drug delivery and biointeractions, making them effective platforms for both direct bacterial killing and potentiation of conventional antibiotics. For instance, catechol-rich hyperbranched poly­(β-amino ester) (HPAE) demonstrated complete inhibition of *E. coli* and *S. aureus*.^26^ In another study, a catechol-based polymer coating codeposited with polyethylenimine (PEI) onto polypropylene membranes achieved 95% antibacterial efficiency against *S. aureus*, while also significantly reducing bacterial adhesion within 24 h.^27^ These studies demonstrate that polymer-based catechol systems maintain the ROS-generating functionality of the catechol moiety while enabling enhanced control over antibacterial agent delivery, site-specific targeting, and biological interface compatibility, all of which are critical parameters for advanced antibacterial applications.

Despite their promising antibacterial performance, catechol-functionalized polymers still face several limitations that can hinder their broader application in biomedical fields. One is safety, which encompasses biodegradability and biocompatibility. In particular, materials intended for biomedical use must degrade safely under physiological conditions without eliciting adverse immune responses or accumulating in tissues.^28–30^ In addition, adequate water solubility under physiological conditions is essential, as it directly influences the biodistribution, cellular interaction, and overall bioavailability.^29^ Next is the stability of catechol moieties within the polymers under the physiological aqueous conditions. The catechol moieties are inherently susceptible to spontaneous autoxidation, even at pH 7.4, which can lead to progressive loss of their functional activity and structural integrity in biological environments.^31–33^ These issues often necessitate protective or stabilizing strategies to preserve functionality and ensure safe, predictable performance in biomedical contexts. Regarding antibacterial activity, the effectiveness of both radical and nonradical ROS as sole agents is limited, as their oxidative properties often fail to achieve the concentrations necessary at the target site for reliable antibacterial effectivity.^17,34,35^ Lastly, the synthesis of some catechol-functionalized polymers involves complex, multistep procedures that are time-consuming, costly and often resulting in limited catechol incorporation and overall reduced yields.

Biodynamers are dynamic polymers formed from biomolecular building blocks via constitutional dynamic chemistry (CDC).^36–42^ In our previous work, we designed biodynamers formed through reversible covalent linkages, specifically imine and acylhydrazone bonds, between amino acid hydrazides and a hexaethylene glycol-conjugated carbazole dialdehyde (CA-HG).^43–46^ Owing to their amino acid-based composition, these polymers are referred to as proteoid biodynamers. A distinctive feature of proteoid biodynamers is their ability to undergo intramolecular self-assemble, primarily driven by π–π stacking interactions between the fluorescent CA-moieties.^38,41,47,48^ This folding process results in compact nanostructures with sizes typically ranging from 3 to 10 nm, classifying them as a subclass of single-chain nanoparticles (SCNPs) formed via CDC.^38,41,48–51^ Also, the inclusion of the HG chains provided a hydrophilic shell to the SCNP, enhancing water solubility, biocompatibility, and amphiphilic character.^44,52^ Besides, in acidic environments, such as bacterial surfaces or microtumor sites, the imine and acylhydrazone bonds cleave, leading to rapid, targeted polymer degradation.^46,53,54^ In previous studies, we demonstrated a variety of versatile applications for proteoid biodynamers, including peptide and nucleic acid delivery, and molecular metal ion sensing.^44,53–56^ Additionally, our recent study on arginine-based biodynamers (ArgBD) demonstrated their effectiveness as antibacterial adjuvants, enhancing colistin-based therapies against Gram-negative bacteria by acting as safe and biodegradable potentiators in antibiotic treatments.^43^

In this study, we designed a DOPA-based biodynamer (DOPA-BD) that integrates catechol moieties with CDC, exploring its potential in biomedical contexts with a focus on antibacterial applications. To this end, l-DOPA-hydrazide (DOPA-Hz) was designed as a catechol-containing monomer inspired by dopamine, serving as the amino acid-derived side chain component in the synthesis of proteoid biodynamers. As a result, the designed DOPA-BD contains catechol moieties in every repeating unit, enabling a high catechol density within the polymer backbone. Moreover, DOPA-BD features a dynamic framework composed of reversible imine and acylhydrazone bonds, which confer biodegradability. It is also expected to self-organize into an SCNP through π–π interactions among CA units, while the HG moieties form a hydrophilic shell, as observed in proteoid biodynamers. These structural features, which are anticipated to provide both biocompatibility and water solubility, are thoroughly studied. This study further investigates the potential of DOPA-BD to enhance the efficacy of conventional antibiotics, with a particular focus on the widely used broad-spectrum macrolide azithromycin (AZM) against an AZM-resistant *E. coli* strain. Overall, DOPA-BD is introduced and investigated as a dynamic and biodegradable platform with potential relevance for future infection-targeted therapeutic applications, offering a potential foundation for further development in antimicrobial strategies.

## 2 Experimental Section

### 2.1 Materials

Dopa-methylester or 3,4-dihydroxyphenylalanine-methylester hydrochloride (C_10_H_13_NO_4_·HCl) and hydrazine hydrate (NH_2_NH_2_·H_2_O) were purchased from TCI, Germany. Methanol (MeOH, CH_3_OH), sodium acetate (CH_3_COONa), and acetic acid (CH_3_COOH) were purchased from Sigma-Aldrich, Germany. For the phosphate buffer (10 mM and pH 7.4), sodium dihydrogen phosphate (NaH_2_PO_4_·2H_2_O) and disodium hydrogen phosphate (Na_2_HPO_4_) were purchased from Fisher Scientific, Germany. For the carbonate buffers, sodium bicarbonate (NaHCO_3_), sodium carbonate (anhydrous) (Na_2_CO_3_), and glutaraldehyde (25%, aqueous solution) were purchased from Sigma-Aldrich, Germany. *N*-Acetylcysteine (NAC), Dulbecco’s Phosphate Buffered Saline (pH 7.4) buffer, and ethanol (absolute) were purchased from Fisher Scientific, Germany. All chemicals were used as received, without additional purification.

### 2.2 General Instrumentation

The ^1^H- and ^13^C NMR spectra were obtained on a Bruker AVANCE III 500 MHz spectrometer, using D_2_O as solvent. For the ^13^CNMR spectrum, the sample was set to a concentration of 20 mg/mL. For the ^1^HNMR spectra, the concentration was set to 6 mg/mL. Spectroscopical analyses were conducted using the Tecan reader spectrometer (Model Infinite M1000, Tecan Austria GmbH, Salzburg, Austria). The Z-Average and Polydispersity Index (PDI) measurements were conducted using the Zetasizer Ultra (Malvern Panalytical, Malvern, UK). The infrared spectrum (IR) of DOPA-BD was measured on an attenuated total reflectance Fourier transform infrared spectroscopy (ATR-FTIR) Vertex 70 spectrometer by Bruker Corporation, Billerica, USA.

### 2.3 Synthesis of DOPA-Hz

For the synthesis of the monomer DOPA-Hz, 3,4-dihydroxyphenlyalanine-methylester hydrochloride (100 mg or 0.404 mmol) was dissolved in 2 mL MeOH. Afterward, 0.157 mL (3.230 mmol, 1:8 molar ratio) of hydrazinium hydroxide (0.485 mL, 10 mmol) was added. The reaction mixture was stirred at room temperature for 24 h under light exclusion. MeOH was then removed under reduced pressure using a rotary evaporator (60 °C, 90 kPa). After evaporating MeOH, the reaction mixture yielded a highly viscous resin with a slight violet color. The product was dissolved in 2 mL water and subsequently lyophilized, resulting in a pink resin product. The resulting product was analyzed by FTIR, ^1^H NMR, and ^13^C NMR to confirm successful product formation. ^1^H NMR (500 MHz, D_2_O): δ = 6.85 (d, *J* = 8.0 Hz, 1H), 6.71 (d, *J* = 2.1 Hz 1H), 6.63 (dd, *J* = 3.4 Hz, 2H), 3.57 (t, *J* = 4.8 Hz, 2H), 2.82 (m, *J* = 5.3 Hz 2H). ^13^C NMR (500 MHz, D_2_O): δ = 173.3, 144.3, 143.2, 128.7, 121.5, 116.9, 166.3, 54.7, 38.9.

### 2.4 Synthesis of DOPA-Biodynamer

For the polymerization reaction, CA-HG was first synthesized following previously reported protocols.^45,46^ DOPA-Hz and CA-HG were then combined in a 1:1 molar ratio (each at 20 mM) in 100 mM acetic acid buffer (pH 5) and stirred under light exclusion for 24 h, resulting in a final polymer concentration of 10 mM. The completion of polymerization was confirmed by ^1^H NMR analysis (0.1 M *d*3-acetate buffer, 500 MHz, D_2_O), made evident by the full depletion of the dialdehyde peak at 9.5 ppm and appearance of a broad peak (4–3.2 ppm).

### 2.5 Molecular Weight (*M*_W_) Analysis

The *M*_W_ of DOPA-BD was analyzed using static light scattering (SLS) on the Malvern Zetasizer Ultra. A dilution series of DOPA-BD was prepared in 100 mM acetate buffer at pH 5. Each sample was then diluted to 2.5, 5, 7.5, 10, and 15 mg/mL and subsequently measured immediately via SLS analysis using the Malvern Zetasizer. The *M*_W_ for each sample set was determined using static Debye plot analysis, and the results were compared. The experiment was conducted in triplicate (*n* = 3). The size exclusion chromatography (SEC) analysis was performed to determine the *M*_W_ distribution of DOPA-BD. The polymer was synthesized in 100 mM acetic acid buffer (pH 5) for 24 h, diluted to 1mg/mL with the SEC eluent, consisting of 80% 0.3 M NaNO_3_ containing 0.01 M NaH_2_PO_4_ and 20% methanol as well as 0.01% LiBr, and allowed to equilibrate for 1 h prior to injection. The measurements were carried out using a Agilent PL aquagel-OH MIXED-M 8 μm, 300 × 7.5 mm column at R.T., with a flow rate of 0.8 mL/min and an injection volume of 20 μL. Calibration was performed using poly­(styrene sulfonate) sodium salt standards (PSS).

### 2.6 Emission Shift upon Introduction of Different pH

To assess the emission shift of DOPA-BD under varying pH conditions, 10 mM of DOPA-BD was synthesized in a cuvette using 100 mM acetic acid buffer. The reaction mixture (200 μL, 0.1 mM) was then analyzed using 96-wellplate UV-STAR by Greiner at different time points (0, 2, 4, 6, 8 and 24 h) via a Tecan reader spectrometer to monitor the emission shift over time (λ_ex_ of 310 nm, a scan wavelength of 2 nm and a gain of 100).

### 2.7 Small-Angle Neutron Scattering (SANS) Analysis

SANS experiments were performed using 10 mM DOPA-BD in 100 mM deuterated acetic acid buffer (pH 5) on the D11 beamline at Institut Laue-Langevin at Grenoble (ILL, France). Two different incident wavelengths λ (6 Å and 11.6 Å) and two sample-to-detector distances (5.6 and 17.6 m) were used, allowing the magnitude of the scattering vector *q* to be varied between 0.002 Å^–1^ and 0.7 Å^–1^. For an elastic process, the magnitude of the scattering vector is defined by $q=\frac{4\pi }{\lambda }\sin\left(\frac{\theta }{2}\right)$ , where θ is the scattering angle. Data were corrected for empty cell scattering, electronic background, detector response and then converted to absolute scale (cm^–1^). Finally, the solvent was subtracted as well as the incoherent background due to the signal of hydrogen atoms.

For polymer solutions, the intensity is generally given by the expression obtained for centrosymmetric objects:

1 $$I\left(q\right)=\frac{1}{V}\frac{d\sigma }{d\Omega }=\varphi \Delta \rho^{2}V_{p}P\left(q\right)S\left(q\right)$$

Where *V* (cm^3^) is the sample volume, $\frac{d\sigma }{d\Omega }$ (cm^2^) the scattering cross-section, φ the volume fraction of monomeric units, Δρ^2^ = (ρ_monomer_ – ρ_solvent_)^2^ (cm^–4^) the difference in scattering length density between polymer and solvent, and *V*_p_ the dry volume of the polymer. *P*(*q*) is the form factor containing information about structure and organization of the scattering objects, and *S*(*q*) is the dimensionless structure factor of the solution linked to interactions between objects. Neglecting virial effects (*S*(*q*) ∼ 1), the weight-averaged mass *M*_W_ of the polymers can be easily obtained by extrapolation of the scattering data to zero-*q* (*P*(*q*) = 1), $M_{w}=\frac{I\left(0\right)\times d\times N_{Av}}{{\Delta \rho }^{2}\times \varphi }$ , with *N*_Av_ the Avogadro’s constant and *d* the monomer density.

The scattering length densities per unit volume of the monomer, ρ_monomer_, and solvent, ρ_solvent_, are determined from their known chemical compositions and given by the following relationship:

2 $$\rho =\frac{\sum n_{i}b_{i}}{1.66\times 10^{-24}\sum n_{i}m_{i}\times v}$$

where *n*_i_ is the number of atomic units i, *b*_i_ (cm) the neutron scattering length of species i with mass *m*_i_ and v=1d the specific volume of the monomer, which was determined previously and taken to be equal to 0.685 cm^3^ g^–1^ (value used for the dimer DOPA-BD, monomer CA-HG, and CA) or the solvent (0.9 cm^3^g^–1^ for deuterated water).^54^ Note that a value of 0.76 cm^3^ g^–1^ was used for DOPA-Hz monomer while a value of 1 cm^3^ g^–1^ was used for the HG moiety alone.

### 2.8 Small-Angle X-ray Scattering (SAXS) Analysis

The SAXS analysis was conducted on 10 mM DOPA-BD in 100 mM deuterated acetic acid buffer (pH 5) using the SWING beamline of synchrotron SOLEIL (Saint Aubin, France). An incident beam with an energy of 12 keV and a sample-to-detector distance of 6.2 m were used, allowing to cover a range of scattering wave vectors *q* between 0.0017 and 0.2 Å^–1^. Successive images obtained for the solvent and samples were captured every second and angularly averaged. For each sample, the spectrum averaged over all frames was then converted to absolute units after subtraction of the pure solvent and the empty capillary. Intensities were scaled using the scattering of glassy carbon standard. In the case of SAXS, the scattering length density is calculated by replacing *b*_i_ with *Z*_i_ *r*_e_ in Equation 2, where *r*_e_ = 2.81 × 10^–13^ cm is the classical radius of the electron and *Z*_i_ is the atomic number of the i^th^ atom.

SANS and SAXS scattering length densities (SLDs) calculated for the biodynamers and solvent are collected in Table 1. For the acetic buffer, the volume fractions of acetic acid and sodium acetate were thoroughly taken into account. It is important to note that in order to obtain a good contrast between the dynamers and the solvent, the neutron scattering experiments were carried out in deuterated solvents. Table 1 clearly shows that neutron scattering is sensitive to the whole biodynamer molecule with significant contrasts between monomeric units and *d*_3_-acetate buffer solvent, Δρ^2^, for both the hydrazide and CA. However, the contrast of the HG pendant is particularly high. On the contrary, X-rays performed with hydrogenated solvents are mainly sensitive to CA groups. The two radiations thus provide complementary information useful for understanding the link between structure and properties.

**Table 1:** Scattering Length Densities (SLDs) and Monomer–Solvent Contrasts, Δρ^2^, Calculated for SANS and SAXS

		Chemical composition	ρ_SANS_× 10^–6^(Å^–2^)	$\Delta \rho_{SANS}^{2}$ ×10^–12^(Å^–4^)	ρ_SAXS_×10^–6^(Å^–2^)	$\Delta \rho_{SAXS}^{2}$ ×10^–12^(Å^–4^)
DOPA-BD		C_36_H_44_N_4_O_9_	2.14	16.56	13.23	13.40
DOPA-Hz		C_9_H_9_N_3_O_3_	2.73	12.11	11.63	4.24
CA-HG		C_27_H_35_NO_6_	1.74	19.98	13.34	14.21
CA		C_14_H_8_N	3.35	8.18	12.93	11.29
HG		C_13_H_27_O_6_	0.44	33.29	9.33	0.06
Acetate buffer	pD 5	CD_4_O_2_/CD_3_CO_2_Na	6.21	-	-	-
	pH 5	CH_4_O_2_/CH_3_CO_2_Na	-	-	9.57	-
Water		H_2_O	–0.56	-	9.47	-
		D_2_O	6.33	-	9.47	-

SANS and SAXS data were fitted using a cylindrical model assuming randomly oriented rods.

$$I(q,\alpha )=\frac{\varphi }{V_{c}}\int_{0}^{\frac{\pi }{2}}F^{2}(q,\alpha )\sin(\alpha )d\alpha +bcgk$$ 3

and

4 $$F\left(q,\alpha \right)=2\Delta \rho V_{c}\frac{\sin\left(\frac{1}{2}qL_{c}\cos\alpha \right)}{\frac{1}{2}qL_{c}\cos\alpha }\frac{J_{1}\left(qr\sin\alpha \right)}{qr\sin\alpha }$$

In this model, α represents the angle between the long axis of the cylinder and the scattering vector *q*, *V*_c_ denotes the cylinder volume, and *bcgk* is a scaling constant related to incoherent background scattering. The model also incorporates the cylinder length (*L*_c_), its radius (*r*), and the first-order Bessel function (*J*_1_), which describes the form factor of cylindrical objects.

### 2.9 Cryo-TEM Measurements

Cryogenic Transmission Electron Microscopy (cryo-TEM) imaging of DOPA-BD was carried out using a JEM-2100 LaB6 microscope (JEOL, Akishima, Japan). A 3 μL droplet of the molecular biodynamer solution (10 mg/mL) prepared in 100 mM acetate buffer (pH 5.0) was applied onto an S147–4 holey carbon film (Plano, Germany). Excess liquid was blotted for 2 s to form a thin film. The sample was then rapidly frozen by plunging into liquid ethane at 108 K using a Gatan CP3 cryo-plunger (Pleasanton, California, USA) and subsequently transferred under liquid nitrogen to a Gatan 914 cryo-TEM holder, which was maintained at 100 K. Imaging was performed under low-dose conditions at an accelerating voltage of 200 kV.

### 2.10 Evaluation of *D*_H_ and PDI at Different pH via DLS

The pH-dependent, dynamic behavior of DOPA-BD was analyzed across a pH range of 5 to 7.4 using each 5 mM (3.38 mg/mL) solutions prepared in appropriate buffer systems: acetate buffer (pH 5), carbonate buffer (pH 6.5), and phosphate buffer (pH 7.4). After preparation, samples were mixed and equilibrated at room temperature, protected from light, and analyzed via DLS 15 min postpreparation. Z-average and PDI were determined using the cumulant method at a backscattering angle of 173°. Each condition was measured in triplicate (*n* = 3).

To evaluate the concentration-dependent behavior of DOPA-BD under physiological conditions, DLS measurements were performed at three different polymer concentrations (3, 1 and 0.512 mg/mL) prepared in PBS at pH 7.4. The lowest concentration corresponds to the MIC applied in the antibacterial assays. After preparation, samples were gently mixed, protected from light, and equilibrated at room temperature prior to analysis. DLS measurements were carried out 15 min postpreparation, and Z-average and PDI were determined using the cumulant method at a backscattering angle of 173°. Each condition was measured in triplicate (*n* = 3).

### 2.11 Degradation Study

DOPA-BD was lyophilized and divided into three batches. Each batch was resuspended in a buffer solution, in particular 10 mM pH 5 acetic acid buffer, 10 mM pH 6.5 and pH 7.4 phosphate buffer, yielding each a final polymer concentration of 1 mg/mL. A 500 μL aliquot from each batch was washed using a 3 kDa Amicon Ultra centrifugal filter (Merck KGaA, Darmstadt, Germany) to remove the released DOPA-Hz monomer for each time point. For each filtration via centrifugation, 2,800 × *g* was applied for 30 min to achieve maximum filtration-volume collection. To quantify the released DOPA-Hz, a method for a reversed-phase high-performance liquid chromatography (RP-HPLC) was developed, utilizing a Thermo Scientific Vanquish HPLC instrument (Thermo Fisher Scientific Inc., Waltham, Massachusetts, USA) equipped with a LiChrospher 100 RP-18 (5 μm) HPLC Column, purchased from Merck KGaA, (Darmstadt, Germany). For the measurement, a gradient was applied, starting with 80% pH 3 phosphate buffer as the polar solvent and 20% acetonitrile as the organic solvent as eluent. Calibration curves were generated using DOPA-Hz as a reference standard at different concentrations, plotting the area under the curve (mAU * min) against the concentration in mg/mL (Figure S7). The release of DOPA-Hz in each batch was analyzed and compared to its initial concentration found in the polymer. The experiment was conducted in triplicate (*n* = 3).

### 2.12 MTT Cytotoxicity Assay

Cell viability of DOPA-BD, DOPA-Hz and CA-HG was evaluated using A549 epithelial adenocarcinoma cells (ATCC ACL107, DSMZ, Braunschweig, Germany) via a Thiazole Blue-based MTT [3-(4,5-dimethylthiazol-2-yl)-2,5-diphenyltetrazolium bromide] assay (Merck KGaA, Darmstadt, Germany). For the assay, 2.0 × 10^4^ cells per well were seeded in a 96-well plate and incubated for 24 h under sterile conditions.^45^ Each concentration (ranging from 31.25 to 500 μg/mL) and corresponding sample treatment were tested in triplicate across three wells and incubated at 37 °C with 5% CO_2_ for both 24 and 48 h time points. The MTT assays were performed according to a previously published protocol.^45^ Negative controls (NC) consisted of cells treated with RPMI medium supplemented with 10% FCS, while Triton X (2%) served as the positive control (PC). The assays were conducted in triplicate (*n* = 3) to confirm reproducibility.

### 2.13 LDH Cytotoxicity Assay

The membrane integrity of A549 cells after treatment with DOPA-BD was assessed by quantifying the release of lactate dehydrogenase (LDH) using the *Invitrogen CyQUANT LDH* Cytotoxicity Assay (Thermo Fisher Scientific, Germany), according to the manufacturer’s protocol. After 24 h of compound exposure in a 96-well plate assay plate, 50 μL of the supernatant from each well (including triplicates) was transferred into a new, flat-bottom 96-well plate. An equal volume (50 μL) of freshly prepared reaction mixture consisting of diluted substrate solution and assay buffer was added to each well and thoroughly mixed. The plate was incubated for 30 min at room temperature under light exclusion. Subsequently, 50 μL of stop solution was added to each well and mixed.

Absorbance was measured at 490 nm with background correction at 690 nm using a Tecan reader spectrometer (Model Infinite M1000, Tecan Austria GmbH, Salzburg, Austria). The cells treated with 2% Triton X-100 served as positive control (PC). Untreated cells in medium were used as negative control (NC). The LDH-based cytotoxicity (%) was calculated using the following formula:

5 $$\mathrm{Cytotoxicity}\left(\%\right)=\left(\frac{\mathrm{LDH}\left(\mathrm{sample}\right)-\mathrm{LDH}\left(\mathrm{NC}\right)}{\mathrm{LDH}\left(\mathrm{PC}\right)-\mathrm{LDH}\left(\mathrm{NC}\right)}\right)\times 100$$

### 2.14 Absorption-Based Stability Study

Absorption measurements were conducted in RPMI 1640 medium (pH 7.4, without phenol red). DOPA-BD and DOPA-Hz were prepared at final concentrations of 0.1 mM and 0.4 mM, respectively, and transferred into a 96-well UV-transparent plate. Spectral analysis was performed using a Tecan plate reader at defined time points (0, 6, 24, 48 and 72 h). Finally, the absorbance was recorded across the relevant wavelength range to monitor time-dependent spectral change.

### 2.15 Stability Study

The stability of DOPA-BD was evaluated in PBS (pH 7.4) over time at room temperature, with samples shielded from light. DLS measurements of Z-average and PDI were performed at 0, 6, 24, 48, 72, 96 and 168 h. To investigate the effect of antioxidants, a parallel analysis was conducted on samples containing *N*-acetylcysteine (NAC) at a 0.5:1 molar ratio with DOPA-BD. Three independent samples were analyzed in triplicate (*n* = 3). Additionally, the stability of DOPA-BD was evaluated in RPMI 1640 medium at a concentration of 512 μg/mL, both in the absence and presence of 10% fetal calf serum (FCS). Samples were prepared under light exclusion and stored at room temperature. The time-dependent stability was subsequently assessed at defined time points (0, 6, 24, 48, 72 and 168 h). At each time point, the samples were visually analyzed for changes in color and aggregation and parallelly analyzed by DLS to determine the Z-average and PDI. All measurements were performed in triplicate using three independent samples (*n *= 3).

### 2.16 Cultivation of Bacteria

The antibacterial activity of DOPA-BD was tested using a drug-resistant *E. coli* DH5α strain (AZMr-*E. coli* DH5α), which harbors the plasmid pLp3050sNuc (Addgene plasmid #122030) encoding the erythromycin resistance gene *ermB*.^57,58^ Its gene product confers the strain resistance to azithromycin. Bacteria were stored as glycerol stocks at −80 °C. Initial cultures were prepared by inoculating 5 mL medium from the glycerol stocks. Bacterial cultures were grown in Luria–Bertani (LB) medium (Carl Roth GmbH, Karlsruhe, Germany) under shaking at 180 rpm at 37 °C (preculture). Prior to each MIC experiment, these precultures were diluted to an optical density of 0.1 at a wavelength of 600 nm (OD_600_) with LB broth and incubated again to reach an OD_600_ of 0.5 (BioPhotometer plus, Eppendorf, Hamburg, Germany) (main culture).

### 2.17 Minimum Inhibitory Concentration (MIC) Assay

To determine the antibacterial activity of DOPA-BD and AZM against the AZMr-*E. coli* DH5α strain, a MIC assay was performed. The microbroth dilution assays were prepared and conducted following the EUCAST guidelines.^59^ Stock solutions of AZM (Apollo Scientific Ltd., Stockport, UK) and DOPA-BD were prepared in absolute ethanol and stored at 4 °C for up to 14 days. A bacterial suspension adjusted to 5.5 × 10^5^ CFU/mL was distributed into the wells of a 96-well microtiter plate containing LB medium. Azithromycin (AZM) was added at concentrations of 2, 4, 8, 16, 32, 64, 128, 256, 512 and 1024 μg/mL per well, while DOPA-BD was tested at concentrations of 8, 16, 32, 64, 128, 256, 512, 1024, 2048 and 4096 μg/mL per well. Control wells included untreated *E. coli* in LB broth and sterile LB medium to monitor growth and background noise, respectively. Plates were incubated without shaking at 37 °C for 24 h. The MIC was identified as the lowest concentration of the test compound that inhibited visible bacterial growth, confirmed by an OD_600_ value approximately matching that of sterile medium (∼0.04). The OD_600_ was measured using the Tecan plate reader following a brief shaking step of 60 s.

### 2.18 Checkerboard Assay

The synergistic interaction between AZM and DOPA-BD was analyzed using a checkerboard microdilution assay in a 96-well flat-bottom microtiter plate with LB medium against the AZMr-*E. coli* DH5α strain.^59^. Here, a bacterial suspension of 5.5 × 10^5^ CFU/mL was added to each well of the plate. For the samples, DOPA-BD was added to each well in concentrations of 8, 16, 32, 64, 128, 256, 512, 1024 and 2048 μg/mL. Similarly, AZM was added to the wells in combination and concentrations of 2, 4, 8, 16, 32, 64, 128, 256 and 512 μg/mL. A growth control, as well as a sterility control containing only LB medium, was added to the plate. The plates were then incubated at 37 °C for 24 h, and bacterial growth was assessed by measuring the OD_600_. The lowest concentrations used for AZM and DOPA-BD, which inhibited bacterial growth, were then assessed, and the fractional inhibitory index (FICI) was calculated using the following equation:^60^

6 $$\mathrm{FICI}=\frac{\mathrm{MIC}\left(\mathrm{combined}\right)}{\mathrm{MIC}\left(\mathrm{AZM}\right)}+\frac{\mathrm{MIC}\left(\mathrm{combined}\right)}{\mathrm{MIC}\left(\mathrm{DOPA}\text{‐}\mathrm{BD}\right)}$$

Here, MIC (combined) represents the minimum concentrations of each compound required in combination to achieve bacterial inhibition. The checkerboard assay was conducted in triplicate (*n* = 3). The heat map was generated from OD_600_ values obtained in the corresponding checkerboard assay, using a color gradient to represent bacterial growth: green indicates no growth (OD_600_ < 0.07), yellow to orange indicates moderate growth (OD_600_ = 0.07–0.19), and red corresponds to maximal growth (OD_600_ > 0.19, up to a maximum of ∼0.32).

### 2.19 Scanning Electron Microscopy (SEM)

To visualize morphological changes of the bacterial membranes of AZMr-*E. coli* DH5α after treatment, the strain was exposed to sub-MIC concentrations of AZM (128 μg/mL) and DOPA-BD (512 μg/mL), both individually and in combination (AZM, 128 μg/mL + DOPA-BD, 512 μg/mL), following the treatment protocol described in Section 2.18. Incubation was carried out for 24 h in PBS at 37 °C. An untreated bacterial suspension served as the NC. After incubation, samples were added to 1.5 mL Eppendorf tubes and centrifuged at 5000 × *g* for 5 min to remove the supernatant. Pellets were washed twice with Milli-Q water and resuspended in 200 μL of 2.5% glutaraldehyde (GA) solution. Each suspension was then applied to separate MICA wafers (PLANO GmbH, Marburg, Germany) and incubated for 1 h at room temperature to allow GA-mediated membrane fixation via cross-linking. Following fixation, samples were dehydrated using a graded ethanol series (30, 40, 50, 60, 70, 80, 90, 96 and 100%), with each step lasting 5 min. The final dehydration step with 100% ethanol was repeated twice for each 10 min. After the removal of the final ethanol solution, the wafers were air-dried overnight. Finally, the dried samples were gold-sputtered using a Quorum Q150R ES sputter coater (GaLa Instrumente GmbH, Germany) and finally imaged using a Zeiss EVO HD15 SEM (Zeiss, Germany) operated at an acceleration voltage of 5 kV.

### 2.20 DCFH-DA ROS Assay

The generation of ROS was assessed and quantified using 2’,7’-dichlorodihydrofluorescein diacetate DCFH-DA, from Sigma-Aldrich, Taufkirchen, Germany, following a protocol adapted from Ravichandiran et al.^61^ The assay was performed using the AZMr-*E. coli* DH5α strain.^58,59^ This strain can be also engineered to heterologously express mCherry as a fluorescent reporter, under the control of a constitutive promoter PtipA.^60^ A bacterial suspension with an OD_600_ of 1.0 was prepared from an overnight preculture in LB medium. From this, 2 mL were aliquoted into each reaction tube, washed twice by centrifugation (5 min at 10,000 × *g*), and resuspended in sterile DPPS (Gibco Invitrogen, Grand Island, USA). The cells were then incubated with DCFH-DA at a final concentration of 10 μM for 30 min in the dark at 37 °C and 180 rpm. Following incubation, unreacted dye was removed by washing the cells twice with sterile DPPS (centrifuging for 5 min at 10,000 × *g* each time). Bacteria were then treated with specified concentrations of dopamine HCl, DOPA-biodynamer, DOPA-Hz, and CA-HG, independently for each 4 and 24 h under the same incubation conditions. Finally, the fluorescence of oxidized DCF was measured using the plate reader (emission wavelength (λ_ex_) of 488 nm, emission wavelength (λ_em_) of 535 nm). Given the structural similarity to the side chain in DOPA-BD and prior studies showing ROS generation, dopamine HCl was selected as the positive control (PC).^25,62^ Untreated bacteria served as negative control (NC). To exclude any interference from the fluorescence of DOPA-BD and CA-HG, background fluorescence at λ_ex_ = 488 nm and λ_em_ = 535 nm was measured and subtracted from the assay results. The ROS assay was conducted in triplicate (*n* = 3).

For the visualization of ROS generation, bacteria exposed to 250 μM dopamine and DOPA-BD, respectively, were imaged after 4 and 24 h using an inverted fluorescence phase contrast microscope, model Keyence BZ-X810 (Keyence Deutschland GmbH, Leipzig, Germany). Twenty μL of the cultures were used in comparison to untreated controls. The DCF fluorescence was measured using a BZ-X GFP filter set (Model OP-87763) with λ_ex_ = 470/40 nm and λ_em_ = 525/50 nm.

## 3 Results and Discussion

### 3.1 Synthesis of DOPA-BD

As the first step in the design of DOPA-BD, the monomer 3,4-dihydroxyphenylalanine hydrazide (DOPA-Hz) was synthesized. Here, 3,4-dihydroxyphenylalanine methyl ester was mixed in a 1:8 molar ratio with hydrazinium hydroxide in MeOH, following the established method as described (Figure 1A).^46^ The ^1^H NMR analysis of DOPA-Hz confirmed complete removal of the methoxy signal at 3.84 ppm, and all ^1^H NMR and ^13^C NMR peaks were assignable to the expected structure (Figure S1), indicating successful synthesis. FTIR analysis further supported these results, showing a broad absorption band between 3600–2400 cm^–1^ and a peak at 1113 cm^–1^, both characteristic for catechol (Figure S2).

**Figure 1:**
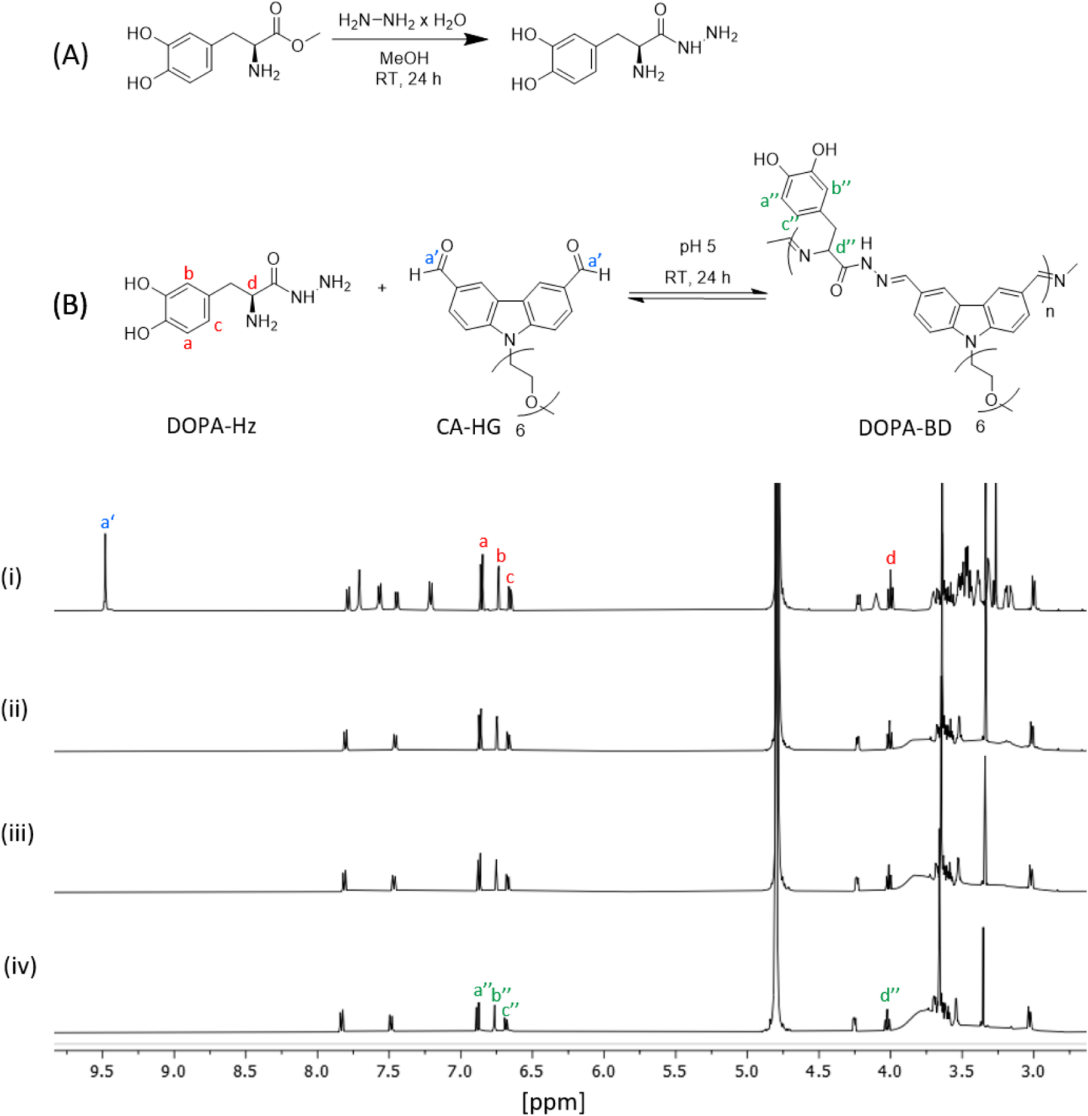
Scheme of two-step synthesis of DOPA-Biodynamers (DOPA-BD). (A) Synthesis of DOPA-Hz via reaction of 3,4-dihydroxyphenylalanine with hydrazinium hydroxide in a 1:8 molar ratio. (B) Polymerization of DOPA-BD through polycondensation under mildly acidic conditions with CA-HG and DOPA-Hz at pH 5. (i)–(iv): ^1^H NMR spectra (64 scans, 500 MHz, D_2_O) of DOPA-BD synthesis measured at (i) 5 min, (ii) 4 h, (iii) 18 h, and (iv) 24 h. Each peak is assigned to the corresponding DOPA-BD structure (a′′–d′′). The depletion of the dialdehyde peak at 9.48 ppm (a′) and the emergence of a broadened peak between 4–3.5 ppm indicates the progression of the polymerization reaction.

In the following step, DOPA-BD was synthesized following the method for previously reported amino acid-derived biodynamers.^45,46^ DOPA-Hz and CA-HG were mixed in a 1:1 molar ratio in 100 mM deuterated acetate buffer (pD 5) and stirred under light exclusion for 24 h (Figure 1B). The progress of the polymerization was monitored by ^1^H NMR at intervals of 5 min, 4, 18 and 24 h Figure 1B­(i–iv). A gradual decrease in the dialdehyde proton signal at 9.48 ppm was observed after 4 h. After 24 h, the signal had fully disappeared, and a broad peak appeared between 3.5 and 4 ppm.

These observations are consistent with previous reports for LysBD, where a polymerization duration of approximately 24 h was required under similar conditions to reach equilibrium of the reaction.^45^ Additionally, as seen in the ^1^H NMR-spectra of Figure 1(iv), the proton signals a′′, b′′ and c′ of the catechol side chain indicated preservation of its reduced state as a catechol group throughout the reaction. Although catechol groups are readily oxidized and unstable in aqueous solution, particularly under basic conditions, their reduced state is maintained under the acidic conditions used during polymerization.^32^ This appears to be due to the pH-dependent oxidation tendency of the dihydroxyphenyl group, with oxidation being mostly suppressed in acidic environments.^31,32^ Overall, the results indicated successful polymerization of DOPA-BD within 24 h, providing the basis for subsequent optical and morphological characterizations.

### 3.2 *M*_W_ Analysis

While NMR spectroscopy offers a useful initial indication of successful polymer formation, determining the *M*_W_ is critical for evaluating the efficiency of the polycondensation reaction and provides an early insight into the elongation behavior of the resulting polymer chains. Evaluating the *M*_W_ of DOPA-BD, first, a SLS analysis was conducted. The polymer was redispersed in acetate buffer (pH 5), filtered, and diluted to a concentration range of 2.5–15 mg/mL. The analysis was immediately conducted, and the *M*_W_ was calculated using a Debye plot analysis (Figure S3).

The SLS analysis revealed an *M*_W_ of 73.16 ± 2.38 kDa, placing DOPA-BD within the established range for amino acid-derived biodynamers (3–138 kDa) and indicating a polymerization efficiency and chain length consistent with those of previously reported biodynamers.^44–46^

To further characterize the polymer metrics, a SEC analysis was performed. The *M*_W_ distribution was determined using UV–vis detection. The analysis yielded a weight-average *M*_W_ (*M*_w_) of 118.2 kDa, a number-average *M*_W_ (*M*_n_) of 41.6 kDa, and a peak *M*_W_ (*M*_p_) of 89.4 kDa. The corresponding dispersity index (PD = *M*_w_/*M*_n_) was 2.84, and the degree of polymerization (DP_n_) was calculated as 61.5, based on the *M*_W_ of DOPA-BD’s repeating unit (676.77 g/mol) (Table S1 and Figure S4). The relatively broad *M*_W_ distribution observed for the SEC method for DOPA-BD is explained by the inherent dynamicity of biodynamer systems and their structural behavior. CA-HG-based biodynamers typically exist as single-chain folded polymers, whose conformational arrangement resembles the secondary structure of peptides. Therefore, *M*_W_ heterogeneity may reflect chain folding, intrachain interactions, and differences in chain extension driven by side chain properties.

Previous studies have shown that the physicochemical nature of side chains in biodynamers strongly influences chain growth and overall polymer morphology.^41,46^ Cationic side chains can promote elongation through cation−π interactions, resulting in extended polymer structures.^46^ In contrast, anionic side chains often lead to electrostatic repulsion, promoting compaction and limiting chain extension, finally leading to oligomer formation or shorter, spherical polymers.^46^ Hydrophobic side chains like phenylalanine are known to induce π–π stacking between aromatic units, which can drive intrachain folding and result in more compact, globular conformations as well.^46^ In the case of DOPA-BD, the catechol side chain is aromatic, hydrophilic, and remains mainly uncharged at pH 5. We hypothesize that this specific combination helps to minimize both repulsive folding and excessive π–π stacking, thus supporting chain extension in solution.^43,45,46^ The *M*_W_ values obtained from both SLS and SEC analyses are consistent with those reported for elongated, rod-like biodynamers such as LysBD and ArgBD. Accordingly, additional structural investigations were conducted later in this study to determine whether DOPA-BD similarly adopts a stacked, rod-like morphology under aqueous conditions.

### 3.3 Emission Shift by Polymerization

CA-HG has been shown to exhibit characteristic changes in optical properties during polymerization and SCNP formation, establishing fluorescence as a sensitive indicator for early structural transitions.^45^ Accordingly, the photophysical properties of DOPA-BD were examined, using changes in fluorescence during polymerization as a preliminary indicator of polymer formation and the possible emergence of SCNP-like structures driven by intramolecular interactions. To monitor the potential structural changes, a time-dependent fluorescence analysis of DOPA-BD was conducted (Figure 2 and Figure S5).

**Figure 2:**
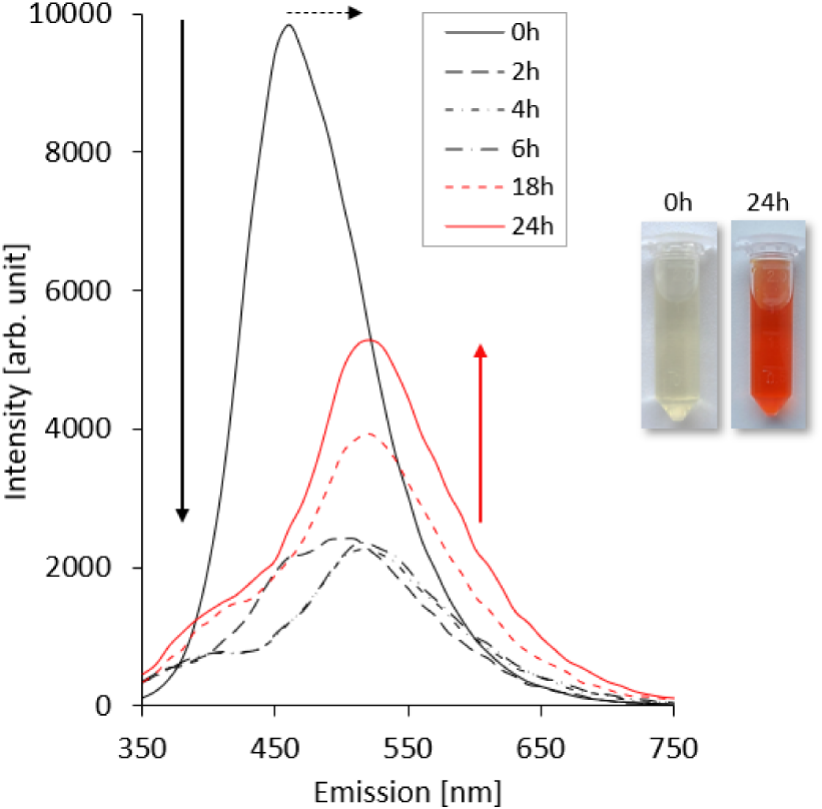
Time-dependent emission changes during the polymerization of DOPA-Hz and CA-HG to form DOPA-BD (0.1 mM). Analysis of emission spectra at defined time points (0, 2, 4, 6, 18, and 24 h), monitoring polymerization progress. The pictures show the color change of the reaction mixture from pale yellow (0 h) to dark red (24 h).

Initiating the polymerization reaction, DOPA-Hz and CA-HG were mixed in equimolar concentrations in 100 mM acetate buffer (pH 5), yielding a 10 mM solution of DOPA-BD. For each time point, an aliquot was diluted to 0.1 mM in phosphate buffer (pH 7.4) to quench the dynamic reaction and allow measurement. Finally, fluorescence emission spectra were recorded at defined time points (0, 2, 4, 6, 8, 18 and 24 h) using an excitation wavelength of λ_ex_ = 350 nm. At 0 h, a strong emission peak at 450 nm was detected, corresponding to the native fluorescence of unpolymerized CA-HG (Figure 2). During the first 4 h, a pronounced decrease in fluorescence intensity and a slight redshift in emission were observed. Between 6 and 8 h, the fluorescence intensity began to recover, accompanied by a continued redshift of the emission maximum, which reached 520 nm after 24 h. Additionally, the reaction resulted in a strong orange colored solution, as shown in Figure 2.

The observed peak shift in fluorescence is likely influenced by the formation of imine and acylhydrazone bonds, which introduce conformational flexibility and dynamic covalent interactions. This time-dependent optical behavior furthermore suggests progressive structural changes during polymerization. Here, the initial quenching is likely associated with π–π stacking between CA cores, while the subsequent partial recovery may indicate rearrangement and chain folding until equilibrium is reached.^41,45,46^ Our observations align with the framework previously described for described for SCNP, in which internal folding and compaction during single-chain formation can be reflected in the emission profile.^50^ A similar trend was reported for the nanorod-like LysBD, where fluorescence changes were associated with SCNP formation by self-folding.^45,46^ In the case of DOPA-BD, these observations support the hypothesis that the polymer undergoes SCNP-like reorganization during polymerization, driven by both dynamic bonding and noncovalent interactions.

### 3.4 Structural Analysis and Morphology

As our preliminary fluorescence data indicated a potential nanorod-like structure of DOPA-BD, we aimed to confirm its solution-state, structure through direct structural analysis using SANS and SAXS and, finally, a cryo-TEM analysis to visualize the polymer’s morphology.

First, SANS measurement was carried out on 10 mM (6.8 mg/mL) DOPA-BD in acetate buffer (pH 5). The scattering profile shown in Figure 3A, which plots the scattered intensity *I*(*q*) as a function of the scattering vector *q*, displays features characteristic of cylindrical structures. At low-*q* values (5 × 10^–3^ to 5 × 10^–2^ Å^–1^), a plateau followed by a Guinier regime was observed, corresponding to the finite *M*_W_ and overall size (radius of gyration, *R*_g_) of the cylindrical objects. This was followed by an intermediate region with an approximate *q* ^–1^ dependence, indicative of rod-like behavior. At higher *q*-values (*q* > 6 × 10^–2^ Å^–1^), a cross-sectional Guinier regime was evident, which transitioned into one or two form factor oscillations that reflect the cross-sectional shape of the cylinders. Also, the cylinders here were quite short as suggested by the narrow *q* ^–1^ regime. The small upturn observed at very small wave vectors indicated a slight tendency toward aggregation at large scales and the formation of a very minor population of aggregates, which can be neglected.

**Figure 3:**
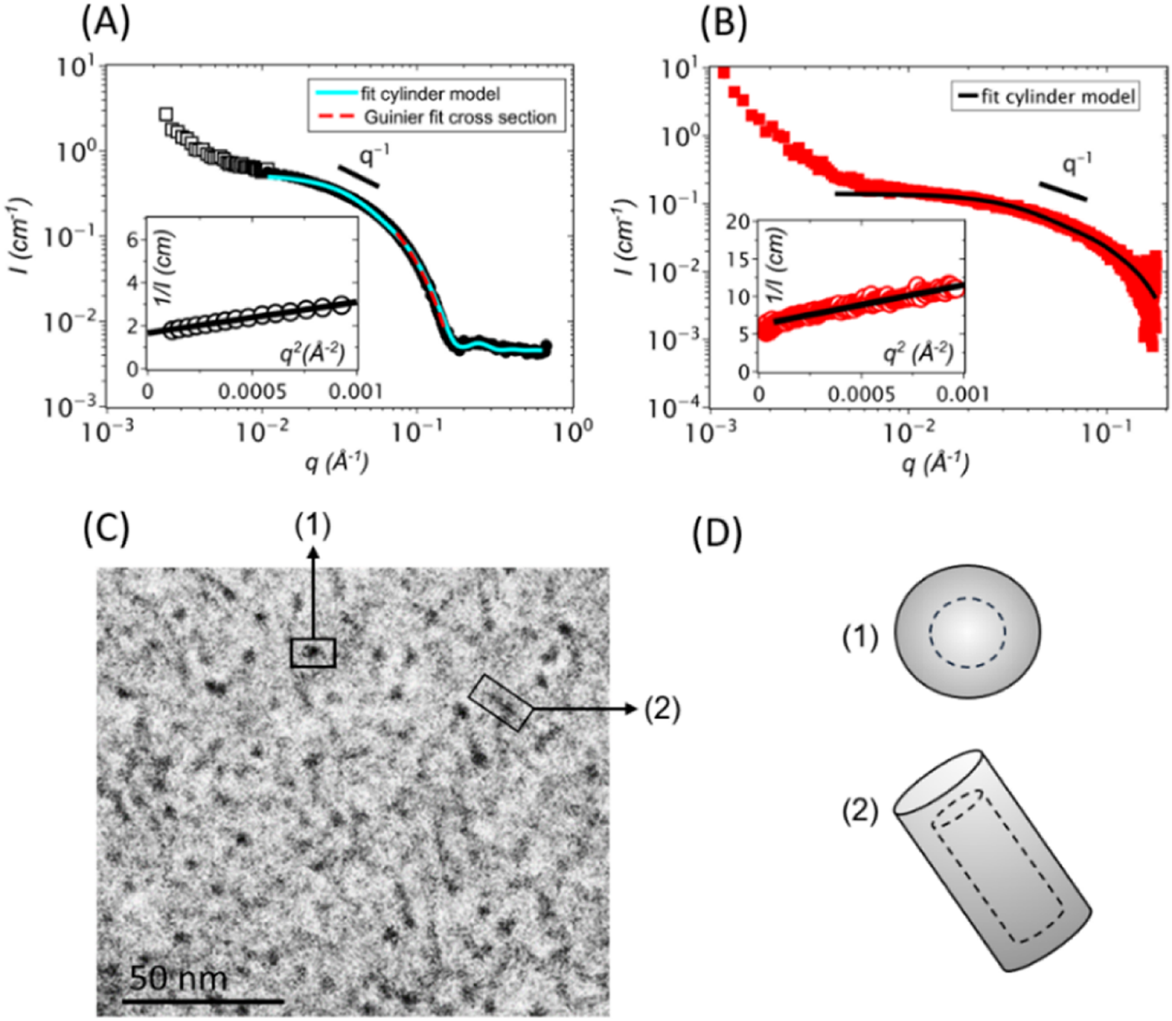
(A) SANS scattering profile of a 10 mM (6.8 mg/mL) DOPA-BD solution in 0.1 M *d*_3_-acetate buffer at pD = 5, fitted in the intermediate/high *q* range with a randomly oriented cylinders model (light blue, see Experimental section) and in the high-*q* range with a Guinier law for the cross-section (red dashed line). Inset: 1/*I* vs *q* ^2^ plot with linear fit in the *qR*_g_ < 1 regime using the Guinier expression $\frac{1}{I\left(q\right)}=\frac{1}{I\left(q^{2}=0\right)}\left(1+q^{2}\frac{R_{g}^{2}}{3}\right)$ . (B) SAXS profile of 10 mM DOPA-BD in 0.1 M acetate buffer with high-*q* to randomly oriented cylinders model (see continuous line). Inset: 1/*I* vs *q* ^2^ plot with linear fit in the Guinier regime. (C) Cryo-TEM image of DOPA-BD, demonstrating the short nanorod-like morphology of DOPA-BD, prepared by mixing the corresponding monomers (10 mM each in *d*_3_-acetate buffer) and allowing reaction at pH 5 for 24 h. (D) Schematic representation of DOPA-BD: (1) coiled top-view and (2) rod-like side-view. The core region (dotted line) is composed mainly of CA units, while the outer shell predominantly consists of HG and DOPA moieties. No stain was used. Image acquisition was achieved at a 2 μm defocus.

A low-*q* Guinier analysis (see inset of Figure 3A) enabled to determine both the radius of gyration *R*_g_ = 51Å and, from *I*(*q* ^2^ = 0), the weight-averaged mass *M*_w_ = 68.5 kDa of the cylindrical structures. This value aligned closely with the *M*_W_ obtained via SLS, which was slightly higher at approximately 73 kDa, likely due to the influence of a minor population of aggregates that contributes more strongly to the lower scattering angles used in SLS. As expected for elongated particles such as cylinders, the *R*_g_ was greater than the hydrodynamic radius (*R*_H_) measured by DLS. From the *R*_g_ value, the contour length (*L*_c_) of the cylinders was estimated using the relation $L_{c}=\sqrt{12}\times R_{g}$ , resulting in a rod-length of approximately 176.7 Å. To obtain more precise structural parameters of DOPA-BD in solution, the scattering data was fitted using a cylindrical model assuming randomly oriented rods, as observed in the cryo-TEM image of Figure 3C, yielding a cylinder length *L*_c_ of 120.4 Å and a radius *r* of 20.3 Å, as shown by the fit in Figure 3A. Although this model provides well-defined dimensional values, additional insight into the internal organization of the cylinders was gained by applying a cross-sectional Guinier analysis in the intermediate *q* range (5 × 10^–2^ to 0.2 Å^–1^). This yielded a cross-sectional radius of gyration (*r*_c_) of 21 Å and a cross-sectional area (*a*_c_) of 555 Å^2^:

7 $$V_{p}P\left(q\right)=\frac{\pi a_{c}}{q}e^{-\frac{r_{c}^{2}.q^{2}}{2}}$$

The cross-sectional area *a*_c_ provides a geometric radius R=acπ of the cylinder’s cross-section, which was found to be 13.3 Å. This value is slightly smaller than the radius of gyration *r*_c_ obtained from the Guinier analysis, a common observation in systems exhibiting core–shell architectures with differing scattering length densities between core and shell regions. The SANS contrast difference is attributed to a carbazole-based core (with a low contrast Δρ_CA core_) and a shell composed of HG and DOPA groups with much higher contrast (Δρ_shell_), as described in Table 1 (Experimental Section 2.8).

To better resolve the shell structure, analytical expressions for hollow cylindrical disks were applied, allowing the determination of the internal (*R*_int_) and external (*R*ext) radii of the nanorod cross-section based on the relations: $a_{c}=\pi \left(R_{ext}^{2}-R_{int}^{2}\right)$ and $r_{c}=\frac{\sqrt{2}}{2}\sqrt{R_{ext}^{2}+R_{int}^{2}}$ The resulting dimensions were *R*_int_ = 18.8 Å and *R*_ext_ = 23.0 Å, indicating a shell thickness of approximately 4.2 Å (Table 2). Since X-rays primarily interact with electron-dense regions, SAXS measurements were conducted to selectively probe the core of the cylindrical structures. As introduced in the Materials and Methods section, the CA core exhibits a significantly higher contrast $\Delta \rho_{CAcore}^{2}$ in water compared to the hydrophilic shell $\Delta \rho_{shell}^{2}$ , rendering the DOPA/HG shell invisible in SAXS. The SAXS scattering pattern (Figure 3B) exhibits similar overall features to the SANS data, with a characteristic sequence of regimes characteristic of cylinder formation. The slight upturn at very low *q*-values appears more pronounced, possibly due to X-rays being more sensitive to electron-dense minor aggregates. Here, a weight-average mass *M*_W_ of 23.5 kDa derived from a Guinier analysis was evaluated. This value is significantly lower than that obtained from SANS, which is expected as SAXS captures only the core signal. Accordingly, the cylinder radius obtained from model fitting is smaller than that from SANS. Resultingly, these differences confirm the formation of fairly short core–shell nanorods by DOPA-BD.

**Table 2:** Structural Characteristics of DOPA-BD at 10 mM (φ = 0.0046) in Acetate Buffer at pH 5 Derived from SLS, DLS, SANS and SAXS

	*R*_g_(Å)	*M*_w_(kDa)	*N*	ρ_cy*l*_×10^–6^(Å^–2^)	*L*_c_(Å)	*r*(Å)	*a*_c_(Å^2^)	*r*_c_(Å)	*R*_int_(Å)	*R*_ext_(Å)
SANS	51.0	68.5	101	3.50	120.4	20.3	555.0	21.0	18.8	23.0
SAXS	-	23.5	-	11.73	119.2	13.3	-	-	-	-
SLS	-	73	-	-	-	-	-	-	-	-

Lastly, cryo-TEM analysis was performed to visualize the morphology of DOPA-BD. As SEM and TEM techniques are not suitable for reliable imaging of biodynamers due to their small size of ∼10 nm, low electron density, and dynamic structure, cryo-TEM provides a more appropriate approach by preserving the hydrated state and enabling visualization. As shown in Figure 3C–D, DOPA-BD displayed both string-like (1) and dot-like (2) structures. These features likely correspond to different orientations of the same rod-shaped particles, specifically side and top views, captured during vitrification. This observation aligns with previous results of similar systems, such as LysBD, where such a dual morphology was attributed to orientation-dependent visualization of elongated nanoparticles.^45^ The rod-like morphology observed for DOPA-BD is consistent with earlier reports on proteoid biodynamers, particularly those forming elongated polymer chains stabilized by tightly stacked π–π interactions and electrostatic forces.^41,45,46^ Beyond their structural stability, rod-shaped nanostructures have been associated with enhanced performance in biological contexts. In particular, their elongated shape can facilitate improved cellular uptake, which is relevant not only for drug delivery but also for antibacterial applications.^52,54^ For example, in our previous study, we showed that the rod-like morphology of ArgBD is related to its interaction with Gram-negative bacteria such as *E. coli*, supporting its role as an antibiotic potentiator.^43^ Similarly, previous studies reported that cylindrical nanoparticles, such as silver-loaded polymeric systems, exhibited greater antibacterial activity than spherical counterparts.^63–65^ This enhanced performance is attributed to more effective membrane contact and increased multivalent interactions, further highlighting the importance of nanoparticle shape in optimizing antimicrobial efficacy.

Taken together, scattering and microscopy data demonstrate that DOPA-BD forms a well-defined single-chain nanorod in solution. This morphology supports our prior hypothesis and suggests favorable interactions with biological systems, positioning DOPA-BD as a shape-controlled, rod-like functional platform relevant to antibacterial applications.

### 3.5 Characterization of *D*_H_ and Dispersity

While polymers are typically characterized by parameters such as chain length and *M*_W_, SCNPs are more accurately described by their degree of internal folding and compaction, commonly assessed via *D*_H_ and PDI. To assess DOPA-BD as SCNP, *D*_H_ and PDI of the polymer were determined using DLS analysis. Measurements were conducted after redispersing DOPA-BD (5 mM) in 10 mM phosphate buffer (pH 7.4) under light exclusion.

The results revealed a *D*_H_ of ∼7.8 nm and a PDI of 0.16, indicating a well-dispersed and narrowly distributed particle population (Figure 4A). To evaluate the concentration-dependent behavior of DOPA-BD under physiological conditions, DLS measurements were performed at three different concentrations (3, 1, and 0.512 mg/mL) in PBS (pH 7.4), including the MIC (0.512 mg/mL), the concentration relevant for the antibacterial assays later in this study (Figure S6). Across all tested concentrations, the *D*_H_ remained consistent at approximately 8 nm, and PDI values remained low (∼0.2). These results indicate that DOPA-BD maintains relatively good colloidal stability, with a consistent *D*_H_ and narrow size-distribution across the tested concentrations.

**Figure 4:**
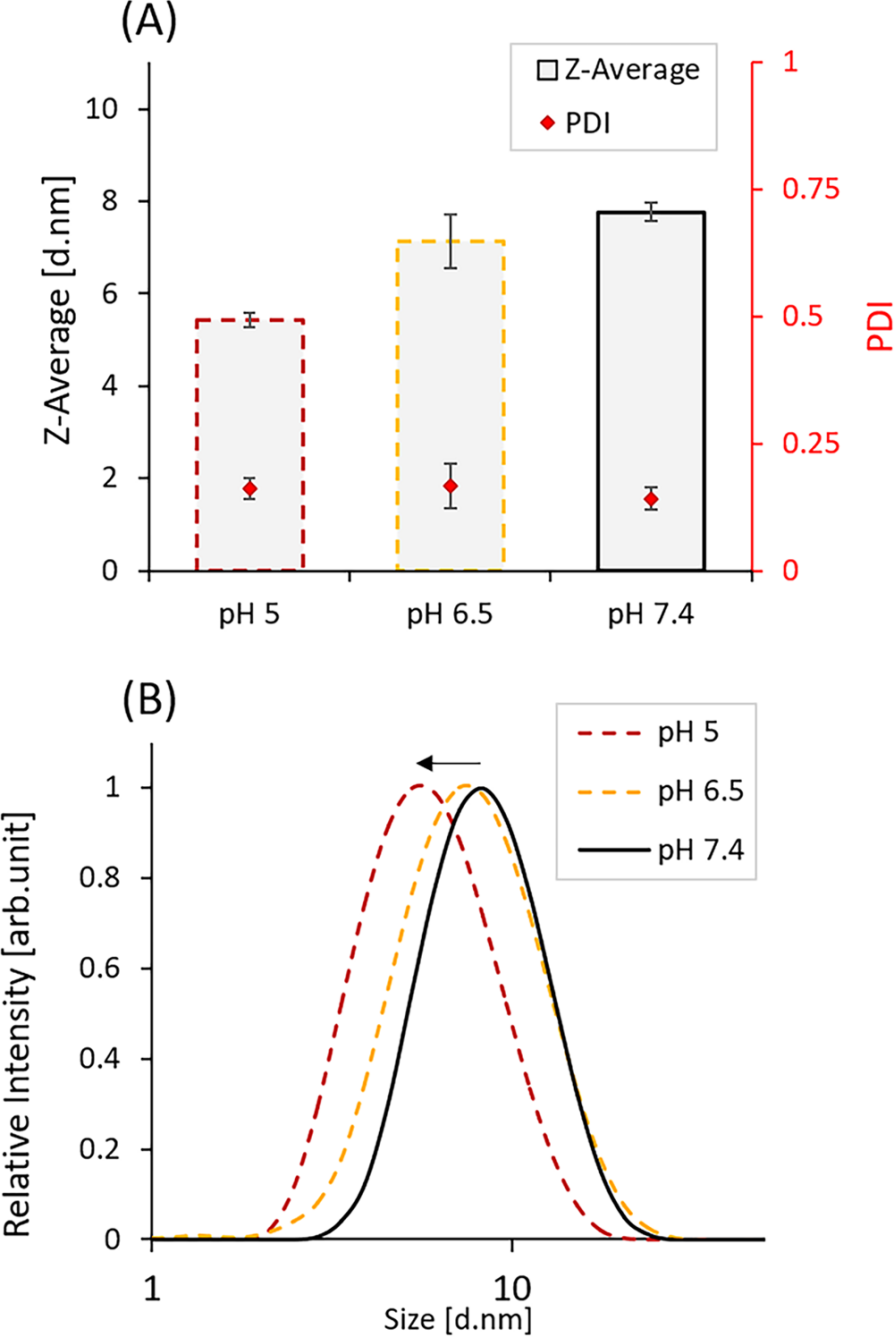
pH-dependent size changes of DOPA-BD analyzed by dynamic light scattering (DLS). (A) Z-average and polydispersity index (PDI) of 5 mM DOPA-BD at pH values of 5, 6.5, and 7.4. (B) Distribution of the hydrodynamic diameter (*D*_H_) of DOPA-BD, showing a decrease in particle size upon a decrease of pH, as observed from the relative intensity profiles. Data represented as mean ± SD: (*n* = 3).

To summarize, the measured *D*_H_ of DOPA-BD are within the expected range for amino acid-derived biodynamers (3–10 nm), as previously reported.^45,46^ The results align with the structural features previously observed by SANS, SAXS, and cryo-TEM analysis, supporting the classification of DOPA-BD as a rod-like SCNP.^50,66^ The combination of moderate *D*_H_ and relatively low PDI reflects a relative uniform particle population with consistent solution-state behavior. Together with the results for the *M*_W_ analysis (∼73 kDa), we were able to confirm a compact and tightly stacked conformation for DOPA-BD.^45^

### 3.6 Effect of pH on *D*_H_ and PDI

After confirming both the morphology and structure of DOPA-BD, we examined how the pH affects the *D*_H_ and PDI of the polymer, since dynamic covalent linkages such as imine and acylhydrazone are known to be pH-sensitive and cleave under acidic pH. Starting the experiment, DLS measurements were conducted on three separate DOPA-BD solutions (5 mM) prepared at different pH values (pH 5, 6.5 and 7.4). Prior to each measurement, samples were incubated for 10 min under light exclusion to ensure complete redispersion (Figure 4 and Table S2). The results showed a clear pH-dependent decrease in *D*_H_, while PDI remained low and stable across all conditions. Specifically, the Z-average decreased from approximately 7.8 nm at pH 7.4 to 5.4 nm at pH 5, corresponding to a size reduction of about 31%. The PDI values ranged from 0.14 to 0.16, indicating that the samples remained relatively monodisperse despite the shift in *D*_H_.

The observed decrease in *D*_H_ at lower pH aligns with the intended pH-responsive behavior of DOPA-BD as a pH-responsive polymer. We hypothesize that this effect is likely a result of acid-induced protonation of dynamic covalent linkages, the imines and acylhydrazones in the polymer backbone, which could facilitate partial bond cleavage and structural destabilization.^41,42,46^ The results reflect the dynamic nature of DOPA-BD’s structure and provide an initial indication of its pH-dependent degradative potential.

### 3.7 pH-Dependent Degradation

Biodegradability is a key requirement for polymer-based materials intended for biomedical applications, as it enables controlled clearance and minimizes the risk of long-term accumulation.^67,68^ To evaluate the pH-responsiveness of DOPA-BD, its degradation was assessed by quantifying DOPA-Hz monomer release at selected time points at pH 5.0, 6.5, and 7.4. The released monomer was analyzed using an absorption-based HPLC method at 280 nm, providing a quantitative measure of polymer breakdown under varying pH conditions (Figure 5, Figure S7).

**Figure 5:**
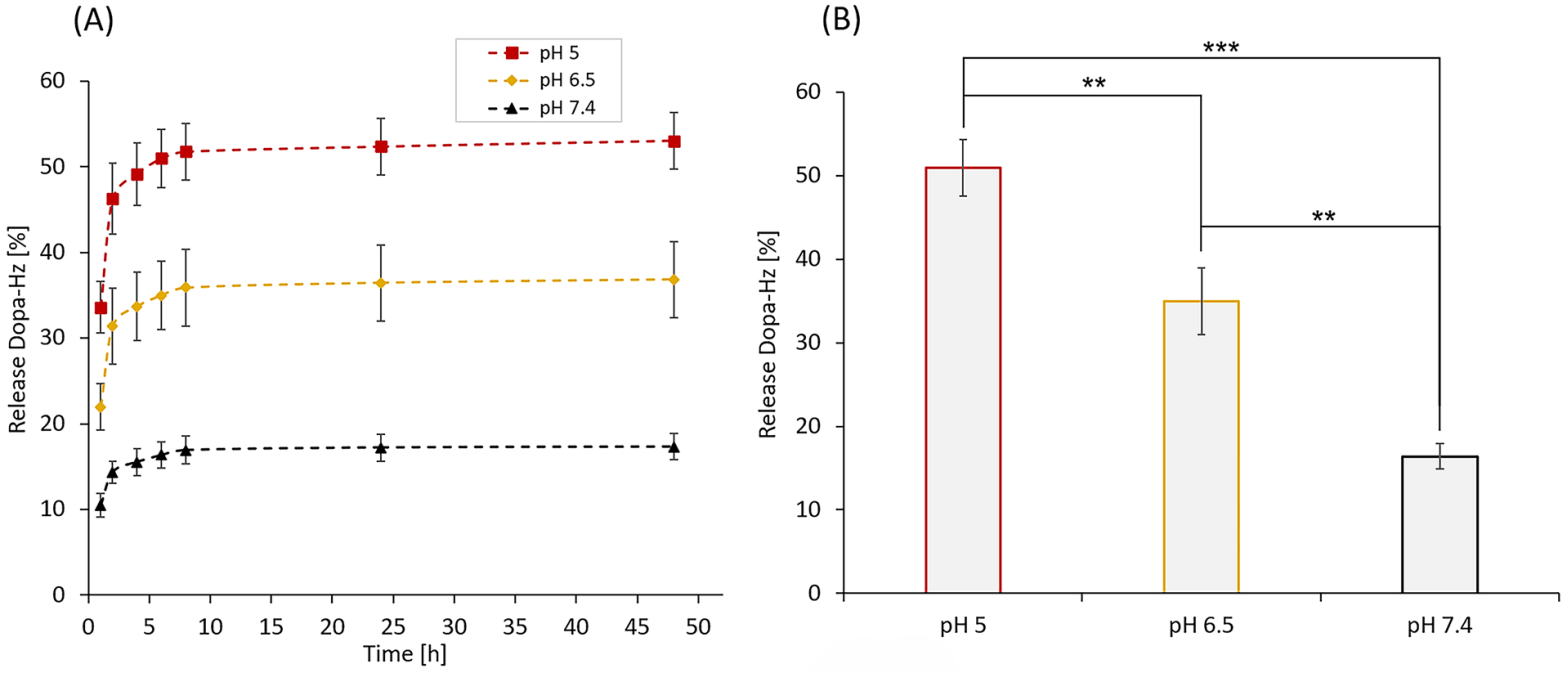
(A) Release profile (%) of DOPA-Hz following pH-dependent degradation of 1 mg/mL DOPA-BD at pH 5 (red), pH 6.5 (yellow), and pH 7.4 (black). After each time point (1, 4, 6, 8, 24 and 48), monomer release was quantified using HPLC (C-18 column, UV–vis detection at 280 nm). (B) Monomer release (%) after 6 h of degradation at each pH value, pH 5.0, 6.5, and 7.4. Data represented as mean ± SD: (*n* = 3). *P*-values were calculated via ANOVA, followed by a Bonferroni post hoc test: *p* < 0.05 (*), *p* < 0.01 (**), and *p* < 0.001 (***). Data represented as mean ± SD: (*n* = 3).

DOPA-BD was incubated in buffer at the respective pH values. At defined time points (1, 2, 4, 6, 8, 24 and 48 h), samples were filtered to separate the released DOPA-Hz. The filtrates were analyzed by HPLC to determine the cumulative release of DOPA-Hz over time. At pH 5, degradation occurred rapidly, with 37% release after 1 h and 58% after 48 h. At pH 6.5, the release reached 24% after 1 h and 39% after 48 h. In contrast, degradation at pH 7.4 was slower, with only 11% release after 1 h and 17% at 48 h (Figure 5A and Table S3). Notably, the release at all pH conditions reached a plateau after 6 h, with final values of 51% at pH 5, 36% at pH 6.5 and 16% at pH 7.4 (Figure 5B).

The observed degradation profile is consistent with our previous findings and supports the intended design of DOPA-BD.^38,42,45,46^ The results clearly demonstrate pH-responsive degradation, with a significantly accelerated degradation of the polymer under mildly acidic conditions at pH 6.5. The results support our hypothesis of a pH-dependent backbone-cleavage and provide direct evidence that DOPA-BD successfully combines catechol functionality with pH-responsive degradability, highlighting the polymer’s potential for applications that require selective breakdown, e.g., acidic microenvironments including inflamed or infected tissues.

### 3.8 Biocompatibility

Next to their biodegradability, polymeric materials intended for biomedical use require careful assessment of biocompatibility, since material-cell interactions and released degradation products can potentially trigger cytotoxic or inflammatory responses. Early in vitro analyses therefore provide a first indication of concentration-dependent safety before proceeding to more advanced studies. Accordingly, MTT assays were performed using mammalian A549 epithelial carcinoma cells to evaluate the biocompatibility of DOPA-BD and its degradation product, DOPA-Hz. The cell line was selected as it represents a well-established in vitro model of human alveolar type II epithelial cells and are widely used for cytotoxicity assessment of drugs and biomaterials intended for lung disease-related applications, including bacterial infections.^69–72^ In this experiment, the cells were exposed for 24 and 48 h to increasing concentrations of both compounds (31.25, 62.5, 125, 250 and 500 μg/mL), and the remaining cell viability was subsequently evaluated (Figure 6).

**Figure 6:**
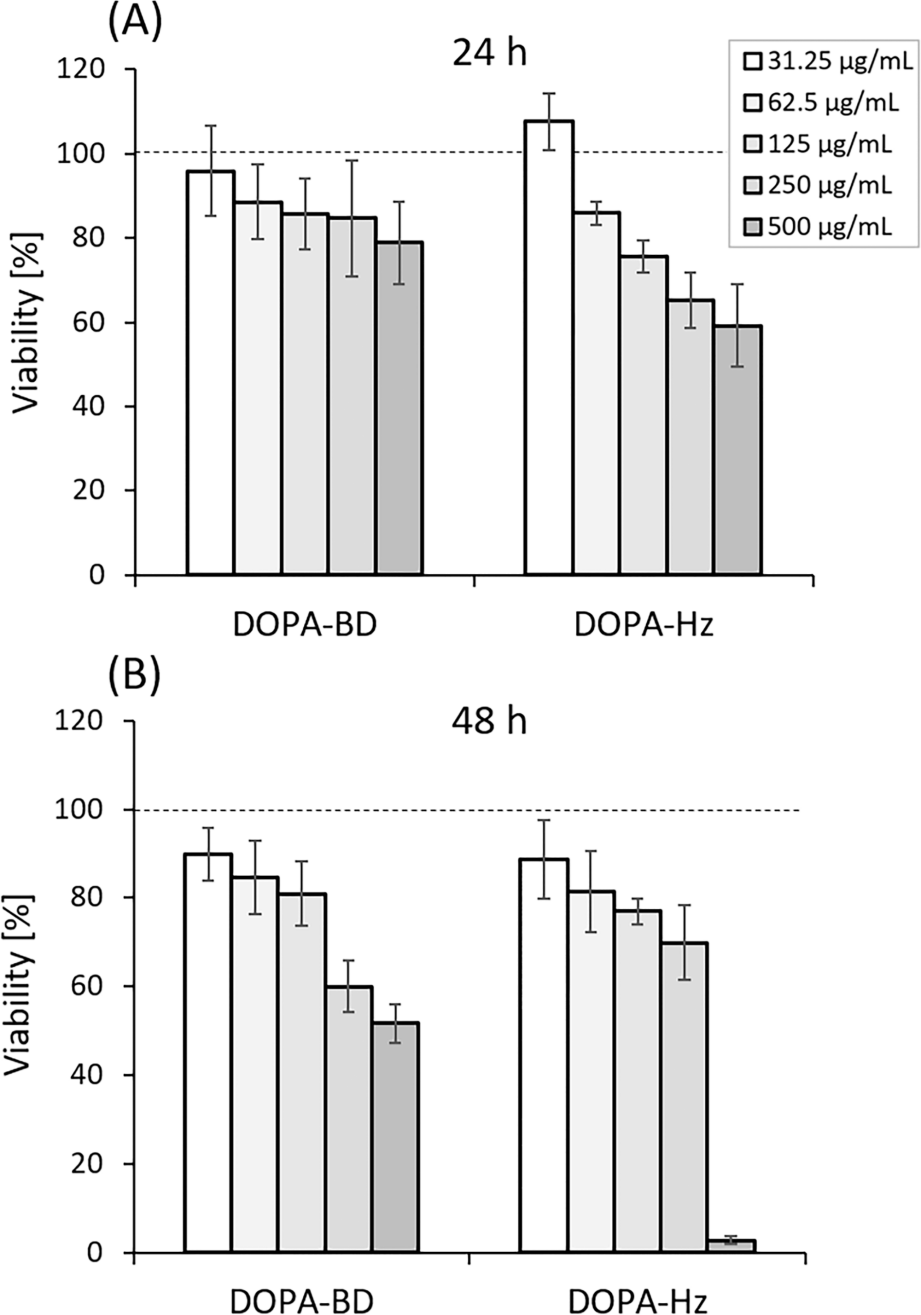
Cell viability study of DOPA-BD and DOPA-Hz at different concentrations at (A) 24 h and (B) 48 h. The MTT assays were conducted using the A549 cell line. Each sample was treated at the specified concentration. Data represented as mean ± SD: (*n* = 3).

At 24 h (Figure 6A), both DOPA-BD and DOPA-Hz maintained high cell viability (>88%) at the two lowest concentrations of 31.25 and 62.5 μg/mL. As concentrations increased, a clear concentration-dependent decrease in viability was observed. DOPA-BD exhibited only moderate cytotoxic effects, maintaining a ∼80% viability at 500 μg/mL, whereas DOPA-Hz reduced viability more substantially to ∼66% at the same concentration.

After 48 h (Figure 6B), the concentration-dependent trend remained, but cytotoxicity became more pronounced. At 500 μg/mL, DOPA-BD retained approximately 51% cell viability, whereas DOPA-Hz showed a markedly different response, with viability dropping sharply to below 3% at the same concentration. The increased toxicity of DOPA-Hz at its highest concentration likely reflects its elevated molar dose of the catechol moiety, potentially producing ROS.^16^ At 500 μg/mL, DOPA-Hz delivers approximately 2.37 mM of active catechols while DOPA-BD can only deliver approximately 0.78 mM. Given that ROS generation is concentration dependent, DOPA-Hz is hypothesized to produce a substantial increased amount of ROS, impacting cell viability.^73^ For CA-HG, the results revealed a concentration-dependent decrease in cell viability, with values remaining around 80% at concentrations up to 125 μg/mL for both 24 h and 48 h (Figure S8). While DOPA-BD at 500 μg/mL contains up to ∼351.84 μg/mL CA-HG, the monomer is covalently integrated and expected to be released only gradually under degradation conditions. Therefore, although the monomer shows comparably increased toxicity as a free compound in comparison to DOPA-Hz, CA-HG is not anticipated to significantly limit the biocompatibility of DOPA-BD in future applications.^53^

The results of this study suggest that DOPA-BD demonstrates sufficient initial biocompatibility within 24 h, even at its highest, tested concentration. These results are highly promising, given the known redox reactivity of catechol groups and therefore potential cell-toxicity for a catechol-containing material. The observed drop in viability to ∼51% after 48 h at its highest concentration of 500 μg/mL indicates a clear time-dependent toxicity effect. This observed cytotoxicity may be related to gradual polymer disassembly, increased exposure of the reactive catechol units, or an accumulation of ROS over time. Another factor could be mitochondrial impairment. Recently, catechol oxidation products have been reported to disrupt mitochondrial membrane potential and ATP synthesis in epithelial cells, leading to increased toxicity.^62^ Importantly, unlike DOPA-Hz, DOPA-BD appears to buffer the acute effects typically associated with catechol moieties to a certain degree. One possible explanation is the presence of the HG chains in the polymer, which, as explained earlier, forms a hydrophilic shell around the polymer.^74^ PEGylation is well-known to reduce cytotoxic interactions by sterically shielding reactive groups and enhancing biocompatibility through increased hydrophilicity and reduced protein adsorption on nanoparticle surfaces.^74^

To provide a first indication of the enhanced stability of DOPA-BD in comparison to DOPA-Hz, UV–vis absorption analysis was conducted over a 72 h period in biorelevant RPMI medium at pH 7.4 (Figure S9). While the absorption profile of DOPA-BD remained largely unchanged, DOPA-Hz exhibited a progressive shift in its spectral features between 6 and 24 h, particularly a decrease and broadening of the peak at 280 nm, consistent with catechol oxidation to quinone species (Figure S9B).^75^ These results provide preliminary support for the theory that polymerization could enhance the oxidative stability of DOPA-BD under biorelevant conditions. However, further validation through detailed and quantitative studies are needed to fully confirm this hypothesis.

Considering the established role of oxidative stress in compromising membrane integrity, the LDH release was evaluated for DOPA-BD as a complementary indicator of cytotoxicity within 24 h of treatment. This approach is consistent with our previous study which employed LDH assays to assess membrane damage in pulmonary cell lines exposed to redox-active compounds.^58^ In our study, the LDH-release assay was performed using A549 cells after 24 h of exposure to DOPA-BD in the same concentration range (31.25–500 μg/mL) applied for the MTT assay (Figure S10). Across all concentrations tested, cytotoxicity remained relatively low and below 20%, with the exception of the highest concentration (500 μg/mL), where an average cytotoxicity of approximately 24% (±4%) was observed.^76^ This result aligns well with the MTT-based viability data, which showed ∼80% cell viability at the same concentration and time point. While catechol-based systems are often associated with ROS-induced membrane damage, the LDH data suggests that DOPA-BD does not cause pronounced membrane disruption under the tested conditions.

Taken together, our results support the classification of DOPA-BD as a biocompatible polymer within defined time and concentration ranges, highlighting the importance of evaluating both short- and longer-term effects on the cell viability of catechol-rich systems.

### 3.9 Stability of DOPA-BD

The long-term stability of DOPA-BD under physiological conditions was investigated to determine whether the polymer maintains its colloidal integrity at physiological pH over time. For this purpose, a time-dependent DLS analysis of DOPA-BD was conducted in PBS buffer (pH 7.4) over a 7-day period (168 h). The analysis was performed at defined time points (0, 6, 24, 48, 72, 96 and 168 h), tracking changes *D*_H_, PDI, and relative scattering intensity (derived mean count rate, DCR).

Within the first 72 h, DOPA-BD remained stable, with a *D*_H_ of ∼8 nm and a PDI of ∼0.15, suggesting uniform particle size and comparable monodispersity (Figure 7A). However, at 96 h, *D*_*H*_ increased to ∼13 nm, accompanied by a 2-fold rise in DCR (Figure 7A, Figure S11). By 168 h, all three parameters had increased markedly: *D*_H_ reached ∼50 nm, PDI rose to ∼0.5, and DCR increased ∼3.6-fold. These changes coincided with a visible color shift from pale yellow to reddish-brown. The results indicate that aggregation is initiated by oxidative processes, most likely driven by the catechol side chains when exposed to oxygen-rich environments.^4,7,23^ To confirm whether oxidation was responsible for the observed instability, we supplemented DOPA-BD with the antioxidant *N*-acetylcysteine (NAC) at a 0.5:1 molar ratio and repeated the time-dependent study under identical conditions.^77^ As expected, the presence of NAC effectively prevented the changes observed in the untreated samples. At both 96 h and 168 h, the *D*_H_ remained stable at ∼8 nm, PDI values were low (0.13–0.14), and DCR showed minimal variation (∼1.1), with no visible color change (Figure 7B and Figure S11).

**Figure 7:**
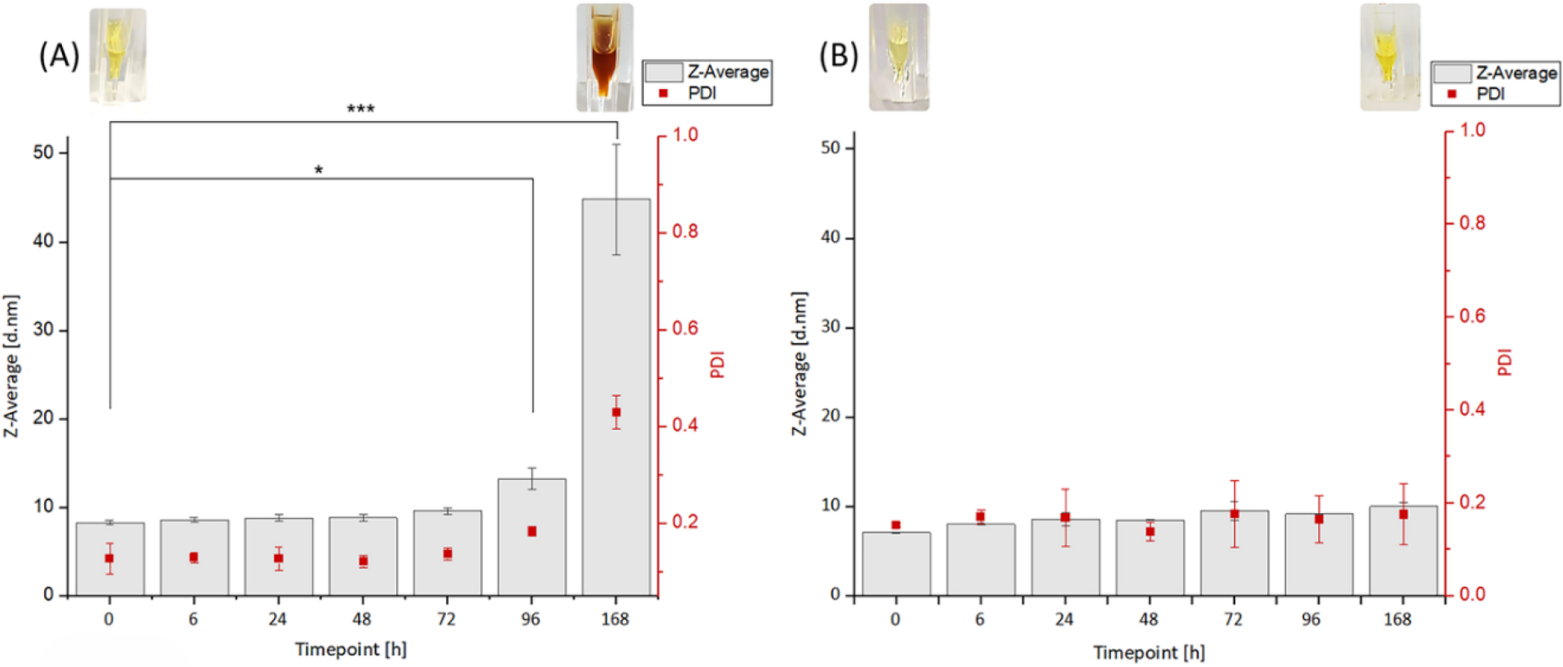
Stability analysis of 5 mM DOPA-BD in PBS buffer, analyzing change in *D*_H_ (Z-Average) and PDI at different time points until 168 h (7 days) via DLS. (A) DOPA-BD in PBS at pH 7.4 under light exclusion. (B) DOPA-BD, with addition of 1:2 molar ratio *N*-acetylcysteine in PBS at pH 7.4 under light exclusion. P-values were calculated via ANOVA, followed by a Bonferroni post hoc test (*p* < 0.05 = significant *, *p* < 0.001= highly significant, ***). Data represented as mean ± SD: (*n* = 3).

To further evaluate DOPA-BD’s behavior under biorelevant conditions, additional stability studies via DLS analysis were performed in RPMI 1640 medium, both with and without 10% fetal calf serum (FCS) (Figure S12). In the absence of FCS, no visible aggregation or color change was observed for up to 72 h, consistent with its stability in PBS (Figure S12A). In the presence of FCS, the solution remained optically stable and clear for up to 48 h, appearing yellowish and without signs of precipitation (Figure S12B). However, at 72 h, a gradual darkening was noted, indicating oxidative changes. By 168 h, both samples, with and without FCS, showed visible aggregation and increased turbidity.

In conclusion, the stability profile of DOPA-BD at physiological pH (7.4) and in biologically relevant media supports its suitability for short-term, solution-based applications. The ability of DOPA-BD to maintain consistent size, dispersity, and scattering intensity over at least 48 h suggests that its molecular design effectively supports colloidal stability in aqueous environments. As discussed previously, this behavior may be attributed to the combination of dynamic covalent linkages and the hydrophilic HG-side chains, which could contribute to solvation and steric stabilization. The observation that antioxidant supplementation with NAC prevented the onset of instability beyond 72 h further supports oxidative degradation as the primary factor contributing to DOPA-BD’s limited long-term stability. To address this limitation, supplementation with alternative antioxidants such as glutathione (GSH) could be explored to further decrease oxidative instability while maintaining biological compatibility.^78^ In addition, temporary protection of the catechol side chains via acetonide formation has been reported as an effective strategy to improve oxidative stability, offering pH-responsive deprotection under mildly acidic conditions relevant to infection sites.^78,79^ Complexation and protection of catechol groups may provide further oxidation resistance through reversible covalent bonding, which can dissociate under acidic conditions.^80,81^ Alternatively, formulation-based approaches, such as encapsulation into stabilizing nanocarriers or hydrogel matrices, may further help overcome the time-dependent oxidative instability of DOPA-BD, offering promising strategies for future therapeutic applications.^82,83^

### 3.10 Antibacterial Potentiation against AZM-Resistant*E. coli*

Antibiotic resistance poses a growing challenge, especially with macrolide-resistant bacterial strains showing diminished susceptibility to the widely used broad-spectrum macrolide antibiotic AZM, leading to potential treatment failures.^84–86^ Thus, following the characterization of DOPA-BD, we evaluated its potential to enhance AZM efficacy against AZM-resistant *E. coli* (AZMr-*E. coli*) through coadministration.

First, we determined the MIC of AZM and DOPA-BD separately by exposing the AZMr-*E. coli* DH5α strain to a range of different concentrations for 24 h. The results showed an MIC of 512 μg/mL for AZM and 2048 μg/mL for DOPA-BD (Table 3). Non-AZM-resistant *E. coli* strains typically show AZM MICs ≤ 16 μg/mL.^87^ The lower sensitivity against AZM of the *E. coli* strain used in this study is explained by the presence of a plasmid carrying the ermB resistance gene, which encodes a methyltransferase that modifies the 23S rRNA binding site, ultimately reducing the binding affinity of azithromycin to the ribosome and resulting in decreased susceptibility.^59,86^ For DOPA-BD, the observed MIC of 2048 μg/mL confirms a lack of severe antimicrobial activity (Table 3 and Figure 8).

**Table 3:** MIC and Checkerboard Assay Results for AZM and DOPA-BD, Tested Individually and in Combination against AZMr-*E. coli* ^a^ ^b^ ^c^

AZM (μg/mL)	DOPA-BD (μg/mL)	FICI	Effect
512	0	-	-
64	1024	0.625	additive
128	512	0.5	synergistic
0	2048	-	-

a A synergistic effect was observed at 128 μg/mL AZM and 512 μg/mL DOPA-BD with a 4-fold decrease in both MIC values (FICI = 0.5).

b All experiments were performed in triplicate (*n* = 3).

c FICI = Fractional inhibitory concentration.

**Figure 8:**
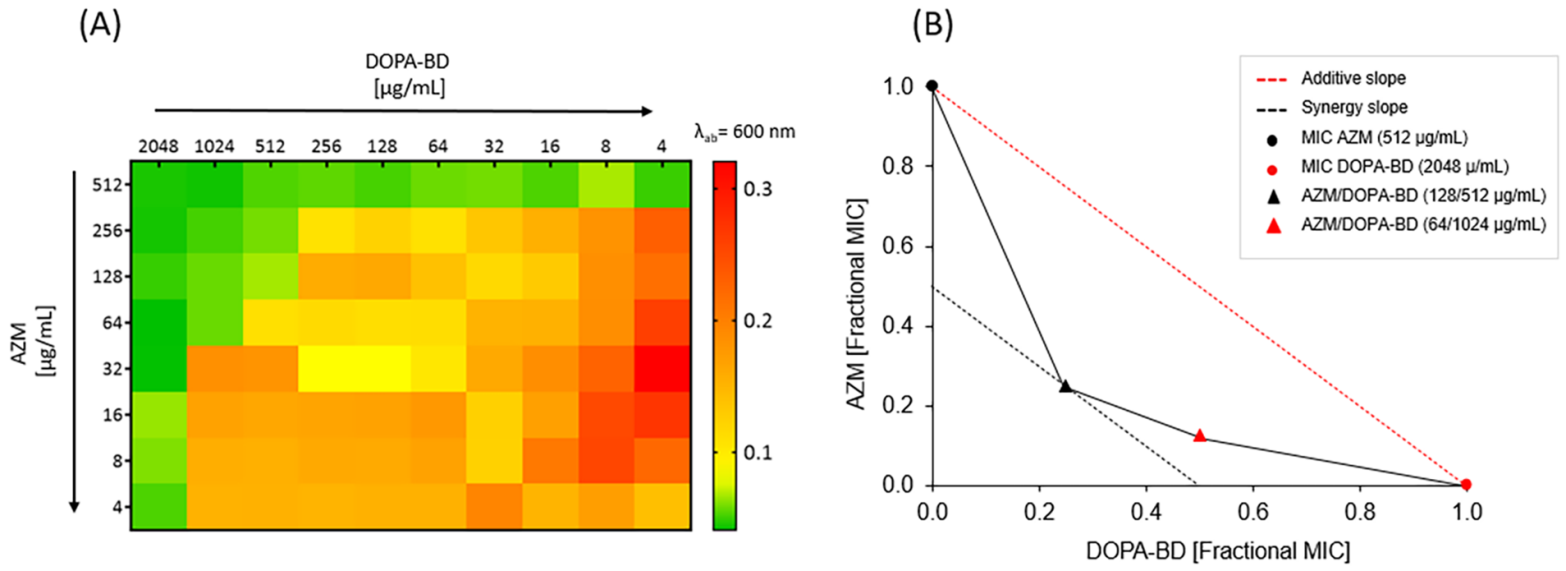
(A) Heat map showing bacterial growth after 24 h for varying concentrations of DOPA-BD (horizontal) and AZM (vertical), based on OD_600_ measurements. The heat map was generated from checkerboard assay data using a color gradient: green indicates no growth (OD_600_ < 0.07), yellow to orange indicates moderate growth (OD_600_ = 0.07–0.19), and red represents maximal growth (OD_600_ > 0.19, up to ∼0.32). (B) Isobologram illustrating the interaction between azithromycin (AZM) and DOPA-BD against *E. coli* DH5α based on fractional MIC values. The red dashed line represents Loewe’s additivity model, where combinations on the line indicate additive effects. Data points below the line indicate synergy. Two effective combinations are shown: 128 μg/mL AZM with 512 μg/mL DOPA-BD (▲), yielding an FICI of 0.5 (synergistic), and 64 μg/mL AZM with 1024 μg/mL DOPA-BD (▲ (red)), with an FICI of 0.625 (additive). The black dashed line illustrates the observed synergy slope based on the measured combinations.

After determining the MICs of both compounds individually, a checkerboard assay was conducted using 2-fold serial dilutions of AZM and DOPA-BD, starting from their respective MICs. The combined antibacterial activity was evaluated across a matrix of concentration pairs by calculating the fractional inhibitory concentration index (FICI). This assay allows for the quantitative assessment of their combined antimicrobial effect, providing insight into potential synergistic interactions.^60^ According to established criteria, FICI values ≤0.5 indicate synergy; 0.5 < FICI ≤ 1 indicates an additive effect; 1 < FICI ≤ 2 indicates no effect; and FICI >2 indicates an antagonistic effect.^60^

The results showed that combining 128 μg/mL AZM with 512 μg/mL DOPA-BD suppressed bacterial growth with a FICI of 0.5, meeting the criteria for synergy (Table 3). This synergistic interaction was reflected by a 4-fold decrease of both MICs. As illustrated in Figure 8, the corresponding heat map (Figure 8A) and isobologram (Figure 8B) position the data point clearly below the additive threshold defined by Loewe’s model, underlining the synergistic interaction. A second but less effective combination, 64 μg/mL of AZM with 1024 μg/mL of DOPA-BD, yielded a FICI of 0.625, indicating an additive effect (Table 3 and Figure 8B).

The checkerboard assay confirmed a synergistic antibacterial effect upon coadministration of DOPA-BD with AZM against the resistant strain. This observed synergy may result from multiple contributing mechanisms. One hypothesis is the involvement of ROS-mediated activity, as redox-active compounds have previously been shown to compromise bacterial defenses and enhance the efficacy of AZM.^58,88,89^ For example, we recently demonstrated that menadione, a well-characterized ROS-generating compound, synergized with AZM to reduce its MIC by 4-fold against an AZM-resistant *E. coli* strain through ROS-induced membrane damage.^58^

### 3.11 Morphological Analysis of *E. coli* following Treatment

To obtain an initial indication of DOPA-BD’s impact on bacterial cell integrity, morphological changes in AZMr-*E. coli* were analyzed by SEM. The bacteria were treated with AZM and DOPA-BD individually at sub-MIC, as well as in combination at their synergistic MIC values (AZM at 128 μg/mL and DOPA-BD at 512 μg/mL). For the negative control (NC), a sample with untreated bacteria was included.

The SEM images revealed treatment-dependent differences in bacterial cell morphology (Figure 9). Untreated and AZM-treated *E. coli* displayed smooth and intact cell surfaces, comprising the outer membrane, cell wall, and cytoplasmic membrane, without notable morphological changes. In contrast, bacteria treated with DOPA-BD at sub-MIC exhibited a tendency toward increased surface roughness, suggesting early signs of membrane stress. The most pronounced morphological changes were observed in the combination treatment at synergistic MIC levels (AZM: 128 μg/mL; DOPA-BD: 512 μg/mL), where a subset of cells displayed visible changes in morphology and irregular shapes, clearly differing from the untreated NC. Similar structural alterations, such as surface lesions and collapse, in *E. coli* following exposure to ROS-generating compounds, were reported in previous studies.^65,90^ As is known, the catechol group in DOPA can exert antimicrobial activity primarily through ROS generation, suggesting a potential redox-based mode of action.^90^ Given the structural similarity of DOPA-BD to other catechol-based systems known to induce ROS, it may have the potential to trigger ROS production in *E. coli*, representing one of the possible mechanisms contributing to the observed synergy.

**Figure 9:**
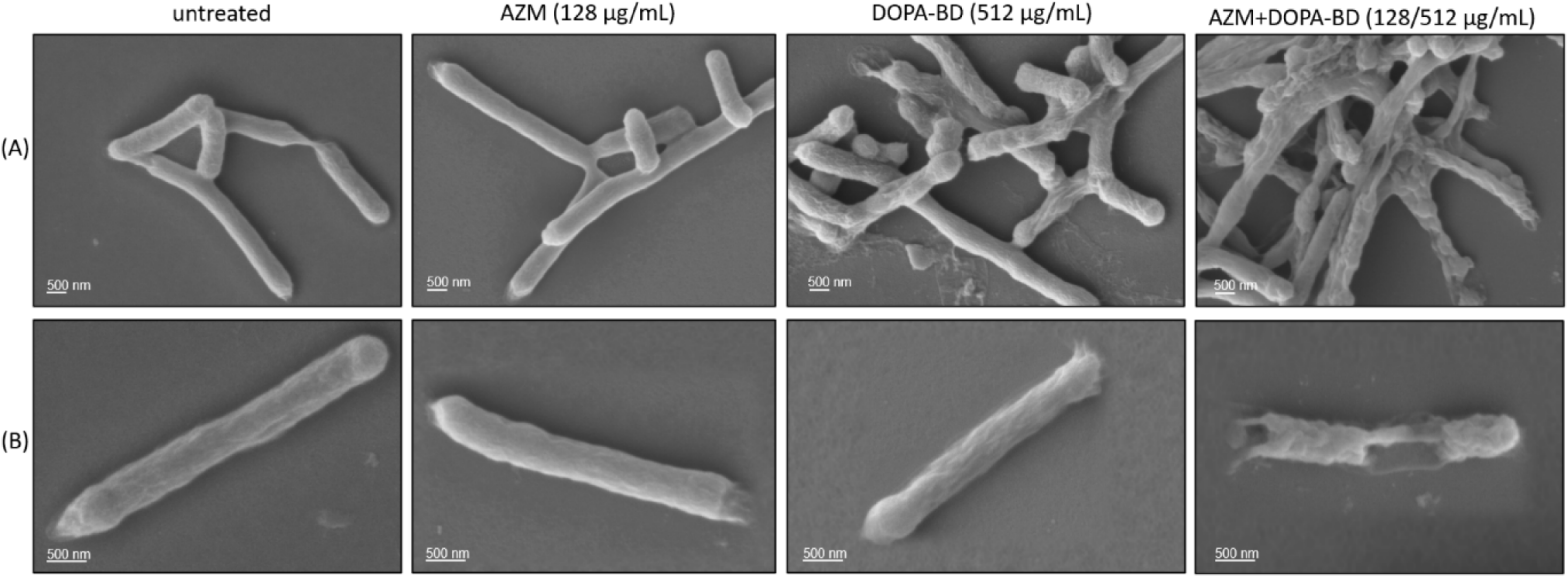
SEM images of *E. coli* after 24 h treatment with AZM and DOPA-BD at sub-MIC concentrations, and their combination at synergistic MIC levels as determined by the checkerboard assay (Table 3). (A) Bacterial cluster and (B) individual bacterium. Images were acquired at 30,000× magnification and 5 kV accelerating voltage.

### 3.12 ROS Formation in AZMr-*E. coli*

As numerous studies have identified ROS generation as a key mechanism contributing to the antibacterial activity of catechol-rich systems, we investigated whether DOPA-BD induces ROS production in the respective bacterial strain. To assess this, a DCFH-DA dye-based assay on AZMr-*E. coli* was performed.^58,62^ The bacteria were treated with DOPA-BD at five different concentrations (7.81–250 μM, which corresponds to 52.9–169.2 μg/mL) for 4 h and 24 h, respectively. ROS levels were quantified by measuring DCF fluorescence intensity, which directly correlates with the amount of ROS produced by the test compounds. Dopamine HCl was included as a positive control (PC) due to its known oxidative activity, and untreated bacteria served as the negative control (NC).

After 4 h, a concentration-dependent increase in DCF intensity was observed across all tested compounds (Figure 10A). Both dopamine and DOPA-BD produced significant amounts of ROS as compared to the untreated control, particularly at the highest tested concentration of 250 μM, with DCF intensities exceeding 5,000 arb. units, representing approximately a 9-fold increase compared to the NC. At lower concentrations (e.g., 7.81 and 15.63 μM), both compounds displayed minimal ROS production, with intensities close to those of the NC. After 24 h, higher ROS levels were detected compared to 4 h, particularly at higher concentrations (Figure 10B), as also visualized by increased fluorescence intensity in Figure 10C. At 24 h and a concentration of 250 μM, DOPA-BD showed the highest level of ROS formation, corresponding to a 3.2-fold increase compared to the 4 h time point after subtracting the DCF intensity of the NC. Dopamine also exhibited substantial ROS production, with a 3-fold increase after subtraction of the NC, indicating oxidative activity comparable to that of DOPA-BD.

**Figure 10:**
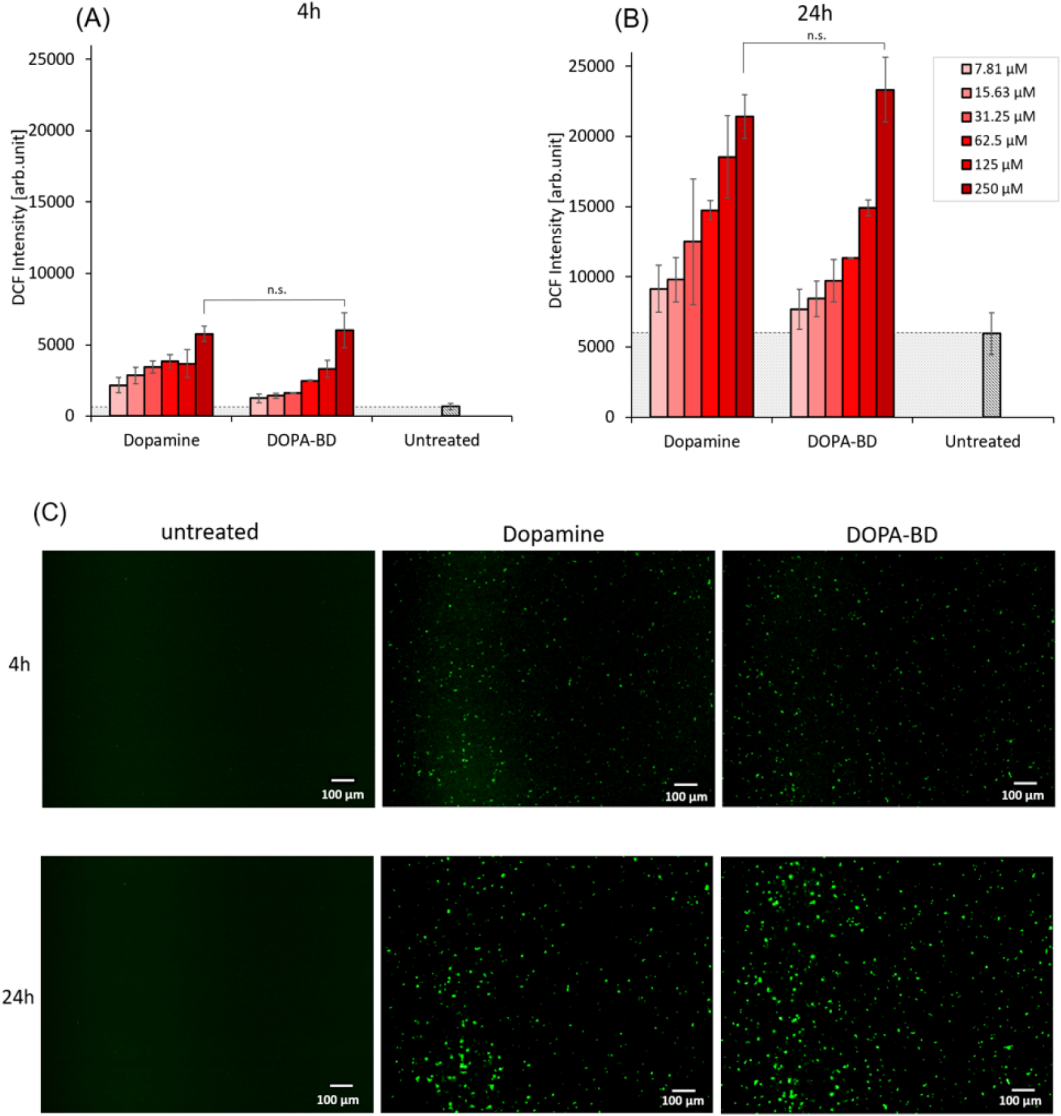
Reactive oxygen species (ROS) generation in *E. coli* induced by dopamine and DOPA-BD, assessed using a DCFH-DA assay at (A) 4 h and (B) 24 h post-treatment. ROS levels are expressed as DCF fluorescence intensity (arb. units) across six concentrations (7.81–250 μM). Dopamine served as PC, while untreated *E. coli* was the NC. Data are presented as mean ± SD (*n* = 3). (C) Visualization of ROS generation at a concentration of 250 μM after 4 and 24 h of treatment. Images were prepared using an inverted fluorescence phase contrast microscope (λ_em_ = 488 nm).

The results confirmed that DOPA-BD induces formation of ROS in the AZMr-*E. coli* strain at 250 μM (approximately 170 μg/mL), a concentration well below the synergistic MIC observed in combination with AZM (512 μg/mL). Quantitatively, DOPA-BD and the PC, dopamine, showed comparable ROS levels at both 4 h and 24 h, with no significant difference at the highest tested concentration of 250 μM (Figure 10).

Although not fully confirmed yet, these results raise the hypothesis that DOPA-BD potentially retains the oxidative reactivity of its catechol-based side chains, which may contribute to the synergistic antibacterial properties through ROS-generation.^7,17,91^ While SEM imaging revealed morphological changes in bacteria treated with DOPA-BD at synergistic concentrations, these observations are interpreted as indicative of general cellular damage rather than direct evidence of membrane disruption. However, such changes can reflect secondary effects associated with oxidative or antibacterial stress, as ROS are known to damage bacteria through multiple mechanisms. One central pathway of ROS-induced bacterial damage involves lipid peroxidation, where membrane lipids are oxidized, compromising the structural integrity of the bacterial envelope.^21,22,92,93^ This can increase membrane permeability, cause leakage of intracellular contents, and lead to cell lysis, often observed as morphological damage such as membrane rupture or collapse. Beyond membrane disruption, intracellular ROS accumulation is also known to disturb bacterial redox homeostasis and damage essential biomolecules, including DNA and proteins.^34^ This oxidative stress may also extend to DNA and proteins, where ROS can induce strand breaks, base modifications, and protein carbonylation, collectively impairing replication, transcription, and essential enzymatic functions.^22,94,95^ Additionally, ROS have been reported to impair efflux pump function, potentially reducing the bacteria’s ability to expel AZM.^88,89^ As a result, ROS-mediated membrane disruption may enhance AZM uptake, allowing it to overcome resistance mechanisms and access its ribosomal target.^58,86,96^

Therefore, the observed synergy between DOPA-BD and AZM against the resistant strains may be associated with the ROS-generating capacity of DOPA-BD. However, further studies are needed to confirm this proposed mechanism of action. Beyond ROS generation, other factors such as the polymer’s inherent toxicity and its interactions with the bacterial membrane may also contribute to the observed effects on cellular morphology. Additional investigations are therefore required to confirm these contributions and to further optimize the system for future application as an antimicrobial potentiator targeting drug-resistant bacteria.

## 4 Conclusions

In this study, we successfully designed and characterized a dynamic, catechol-functionalized biodynamer incorporating CDC via reversible imine and acylhydrazone linkages. DOPA-BD forms a well-defined nanorod-like structure with a *D*_H_ of approximately 7.8 nm and excellent monodispersity, as confirmed by DLS and supported by complementary SANS, SAXS, and cryo-TEM analyses. While its amphiphilic character promotes good water compatibility, the compact SCNP-like morphology may facilitate interactions with bacterial membranes, an important feature for biomedical applications where such structural properties could contribute to enhanced therapeutic efficacy and targeted delivery. DOPA-BD demonstrated good colloidal stability over 72 h under physiological conditions and showed ∼80% cell viability in A549 cells at 500 μg/mL after 24 h, indicating short-term biocompatibility. Its selective degradation under mildly acidic conditions (51% monomer release at pH 5 vs 15% at pH 7.4) further supports its suitability for responsive drug delivery in infection- or inflammation-related environments. When combined with AZM, DOPA-BD significantly enhanced antibacterial efficacy against AZMr-*E. coli*, reducing the MIC of AZM 4-fold from 512 μg/mL to 128 μg/mL, demonstrating its potential to restore antibiotic activity against the resistant strain. Additionally, the polymer generated substantial amounts of ROS at both 4 h and 24 h, reaching levels comparable to dopamine, a well-characterized ROS-producing catechol-containing molecule. Although not yet fully confirmed, the ability of DOPA-BD to induce ROS provides a preliminary indication of a potential ROS-mediated contribution to the observed synergistic antibacterial effect. This hypothesis is further supported by SEM imaging, which revealed bacterial morphology changes consistent with oxidative membrane damage.

Taken together, this study reports DOPA-BD as a biocompatible, redox-active, and biodegradable platform with a defined nanostructure and high catechol density, capable of achieving synergistic antibacterial effectivity in cotreatment with AZM. Given the concentration- and ratio-dependent nature of the observed synergy, future advanced codelivery strategies should be considered to ensure optimal local concentrations of both DOPA-BD and AZM at the site of infection. Approaches such as the coencapsulation of both agents into nanocarriers or the development of AZM-loaded, biodynamer-based pH-responsive nanoparticles may help improve therapeutic efficacy and delivery precision. With further structural optimization, in vivo validation, and comprehensive assessment of its safety profile planned in the future, DOPA-BD holds strong potential as an antibiotic potentiator for future infection-targeted biomedical applications.

## Supplementary Material



## Notes

Spectroscopic characterization of DOPA-Hz and DOPA-BD (^1^H and ^13^C NMR, FTIR, UV–vis); *M*_W_- and dispersity analysis by SEC and SLS; detailed DLS investigations in different buffers and biologically relevant media; calibration and release studies of DOPA-Hz by HPLC; extended cytotoxicity assessments of DOPA-BD and CA-HG (MTT and LDH assays); extended stability analyses of DOPA-BD (PDF)

## Abbreviations

BD: Biodynamer
CA-HG: Carbazole-hexaethylene glycol
DOPA-BD: Dopa-biodynamer
LysBD: Lysine-biodynamer
ArgBD: Arginine-biodynamer
ROS: Reactive oxygen species
AZM: Azithromycin
DCC: Dynamic covalent chemistry
CDC: Constitutional dynamic chemistry
