# Supplementary material for: Design of Poly-Catechol Biodynamers for Potentiation of Antibiotic Efficacy against Drug-Resistant Bacteria
Source: Biomacromolecules. 2026 Feb 23;27(3):1949–68. doi: 10.1021/acs.biomac.5c02130 (PMC12977063; doi:10.1021/acs.biomac.5c02130)
Supplement: Supplementary file 1 [file bm5c02130_si_001.pdf]

Supporting Information

Design of Poly-Catechol Biodynamers for Potentiation of Antibiotic Efficacy against Drug-resistant Bacteria

Lena Zeroug-Metz[1,6], Kristela Shehu[2,3,6], Justine Bassi[4,5,6], Justin Podlecki[1,6], Philipp Sonntag[1,6], Marcus Koch[3,7], Anastasia Christoulaki[8], Eric Buhler[8], Anna K.H. Hirsch[4,5,6], Annette Kraegeloh[3], Marc Schneider[2,6], Sangeun Lee[1,4,6]*

[1]Pharmaceutical Materials and Processing, Department of Pharmacy, Saarland University, Campus C4. 1, 66123, Saarbrücken, Germany

[2]Biopharmaceutics and pharmaceutical Technology, Department of Pharmacy, Saarland University, Campus C4.1, Saarbrücken 66123, Germany

[3]INM – Leibniz Institute for New Materials, Campus D2.2, 66123 Saarbrücken, Germany

[4]Helmholtz Institute of Pharmaceutical Research Saarland (HIPS) – Helmholtz Centre for Infection Research (HZI), Campus E8.1, 66123 Saarbrücken, Germany

[5]Medicinal Chemistry, Department of Pharmacy, Saarland University, Campus E8.1, 66123 Saarbrücken, Germany

[6]PharmaScienceHub (PSH), Saarland University, 66123 Saarbrücken, Germany

[7]University of Applied Sciences, htw saar, Goebenstr.40, 66117 Saarbrücken, Germany

[8]Laboratoire Matière et Systèmes Complexes (MSC), UMR CNRS 7057, Université Paris Cité, Physics Department, Bâtiment Condorcet, 75013 Paris, France

Table of Contents

Figure S1. (A) ^{13}C-NMR and (B) ^{1}H-NMR spectra of DOPA-Hz..3

Figure S2. FTIR spectrum of DOPA-Hz 3

Figure S3. Static Debye plot / M_W determination of DOPA-BD...4

Table S1: SEC of DOPA-BD 4

Figure S4. SEC trace/peak 4

Figure S5. Spectroscopical analysis of DOPA-BD 5

Figure S6. DLS analysis of DOPA-BD 5

Table S2: DLS analysis of DOPA-BD in different buffers...6

Figure S7. Calibration curve of DOPA-Hz via HPLC analysis 6

Table S3: Release of DOPA-Hz in % via HPLC analysis 7

Figure S8. MTT/ Cytotoxicity assays of CA-HG..7

Figure S9. UV-vis absorption spectra of (A) DOPA-BD and (B) DOPA-Hz 8

Figure S10. LDH release assay/ Cytotoxicity assay of DOPA-BD evaluated by a 8

Figure S11. DLS analysis of DMCR: DOPA-BD w and w/o NAC supplementation 9

Figure S12. DLS stability study of DOPA-BD in RPMI medium w and w/o FCS 9-10

References..10

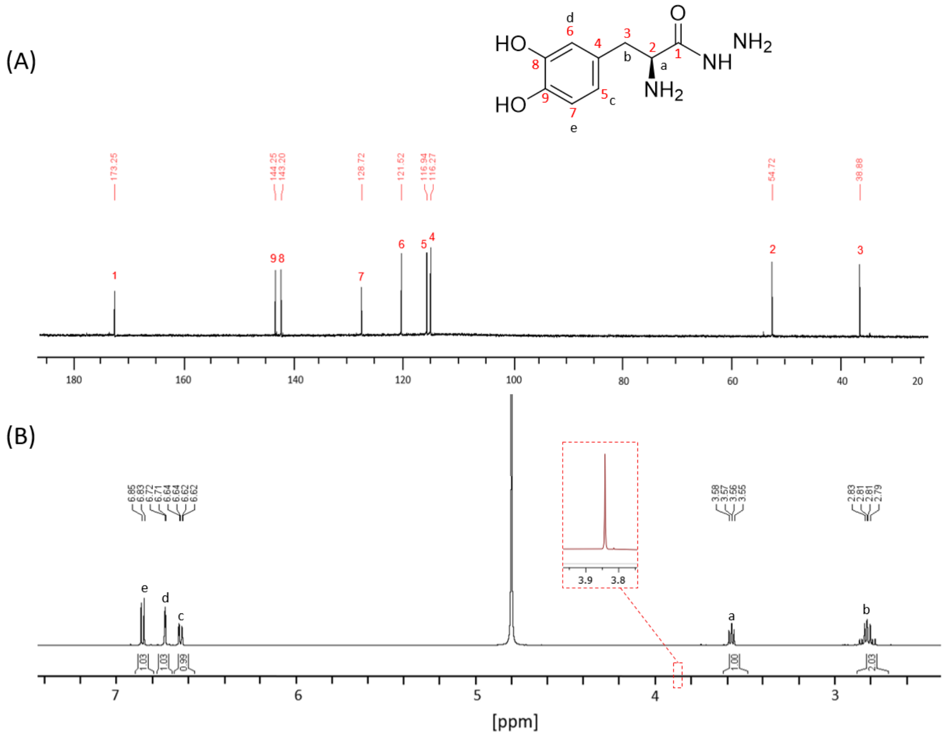

Figure S1. (A) ^{13}C-NMR spectrum (2500 scans) and (B) ^{1}H-NMR spectrum of DOPA-Hz, measured in D_2O via a 500 MHz Bruker instrument. The red box highlights the distinct methoxy signal at approximately 3.84 ppm, corresponding to the starting material 3,4-dihydroxyphenylalanine methyl ester.

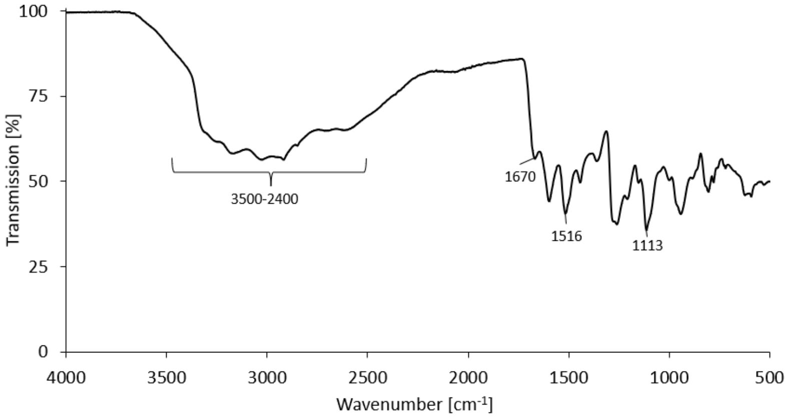

Figure S2. FTIR spectrum of DOPA-Hz showing characteristic absorption bands: a broad peak at 3500–2400 cm^{-1} corresponding to O–H and N–H stretching vibrations, a peak at 1670 cm^{-1} assigned to C=O stretching of the hydrazide group, 1516 cm^{-1} indicating aromatic C=C stretching, and 1113 cm^{-1} corresponding to phenolic C–O stretching vibrations.[1]

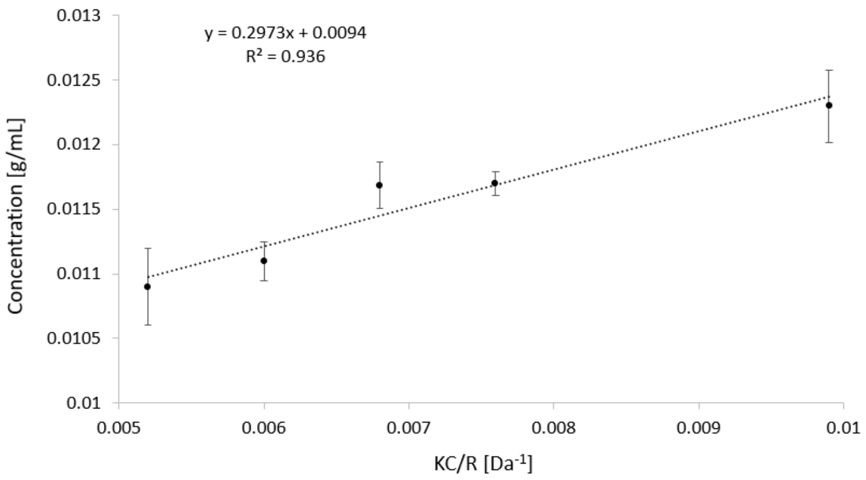

Figure S3. Static Debye plot used to determine the M_W of DOPA-BD in 10 mM phosphate buffer (pH 7.4). Data are shown as mean ± SD (n = 3).

Table S1: Results of the M_W analysis of 1 mg/mL DOPA-BD via SEC**.**

Sample	*Concentration (mg/mL)*	M_w *repeating unit*	*RT*	M_p	M_w	M_n	*PD*	*DP*
DOPA-BD	1	676.77	7.83	89.4	118.2	41.6	2.84	61.5

RT= retention time (min), M_p*= peak molecular weight (kDa),* M_w*= weight-average molecular weight (kDa),* M_n*= number-average molecular weight (kDa), PD = Polydispersity index, DP = Degree of polymerization*

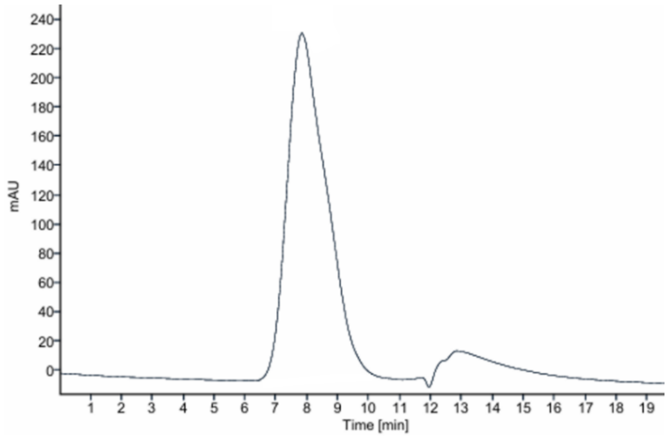

Figure S4. SEC trace/peak of DOPA-BD at 1 mg/mL, measured via UV-vis detection at 254 nm, showing the main elution peak at a retention time of 7.83 min.

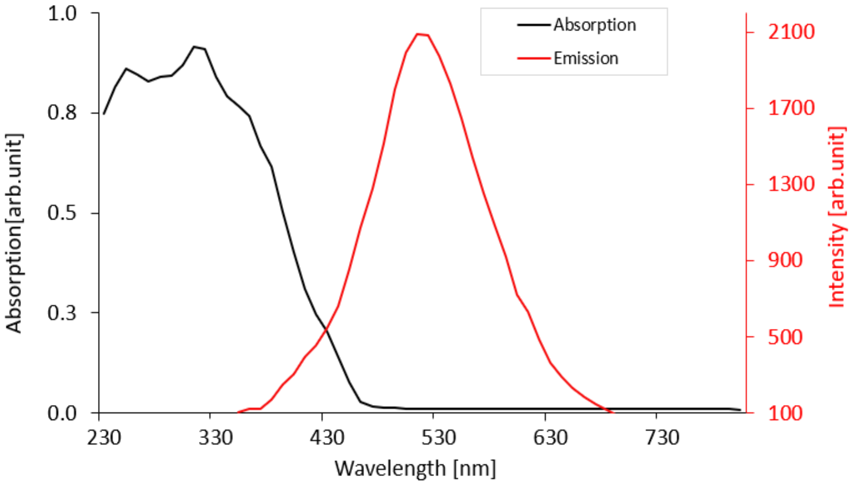

Figure S5. Spectroscopical analysis, evaluating the absorption and emission spectra of 0.1 mM DOPA-BD in phosphate buffer at pH 7.4. The emission was measured at an excitation wavelength of λ_{ex}= 350 nm.

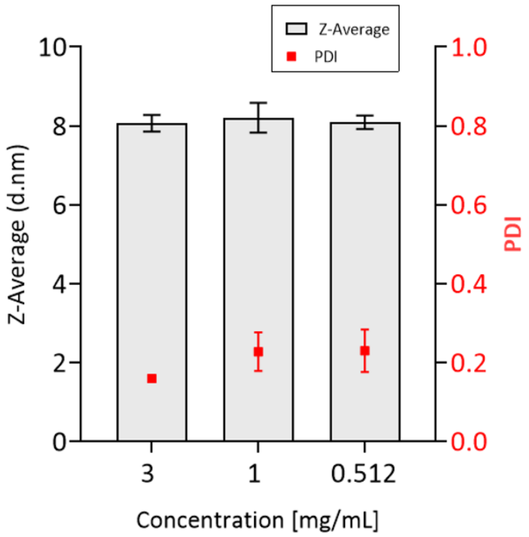

Figure S6. DLS analysis of DOPA-BD at different concentrations (3, 1, and 0.512 mg/mL) in 10 mM PBS buffer at pH 7.4. Data are presented as mean ± SD (n = 3).

Table S2: Results of the DLS analysis of 5 mM DOPA-BD in different buffers at a concentration of 10 mM**.** Data represented as mean ± SD:(n = 3)

pH	*Z-Average (d.nm)*	*PDI*
5	5.44 ±0.16	0.16 ± 0.02
6.5	7.08 ± 0.48	0.17 ± 0.04
7.4	7.77 ± 0.19	0.14 ± 0.02

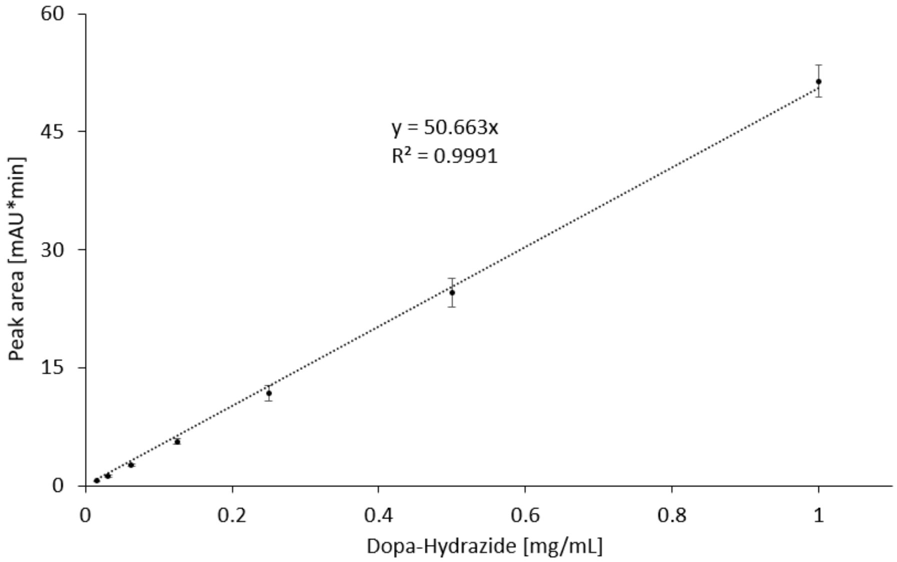

Figure S7. Calibration curve via HPLC analysis of DOPA-Hz plotting concentration (mg/mL) against peak area (mAU*min), used for quantifying DOPA-Hz release during the degradation analysis. Data are presented as mean ± SD (n = *3*).

Table S3: Release of DOPA-Hz in % via HPLC analysis calculated from 1mg/mL (500 µL) DOPA-BD in the respective buffer solutions. Data are presented as mean ± SD (*n* = 3)

Timepoint [h]	Release [%] **pH 5**	Release [%] **pH 6.5**	Release [%] **pH 7.4**
1	37 ±6	24 ±4	11 ±1
2	46 ±4	31 ±4	14 ±1
4	50 ±3	34 ±3	16 ±2
6	51 ±2	36 ±4	16 ±2
8	52 ±3	36 ±4	17 ±2
24	57 ±8	38 ±4	17 ±2
48	58 ±9	39 ±6	17 ±2

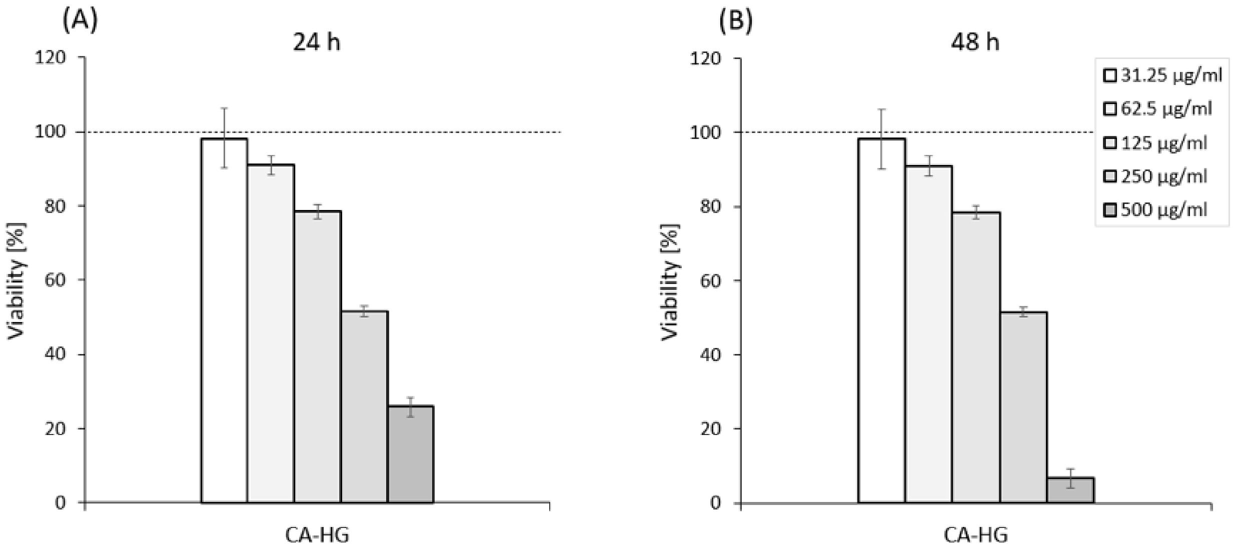

Figure S8. Cytotoxicity of CA-HG evaluated by MTT assays on A549 cells after 24 and 48 h of treatment across a concentration range from 31.25 to 500 µg/mL. Data represented as mean ± SD (*n* = 3).

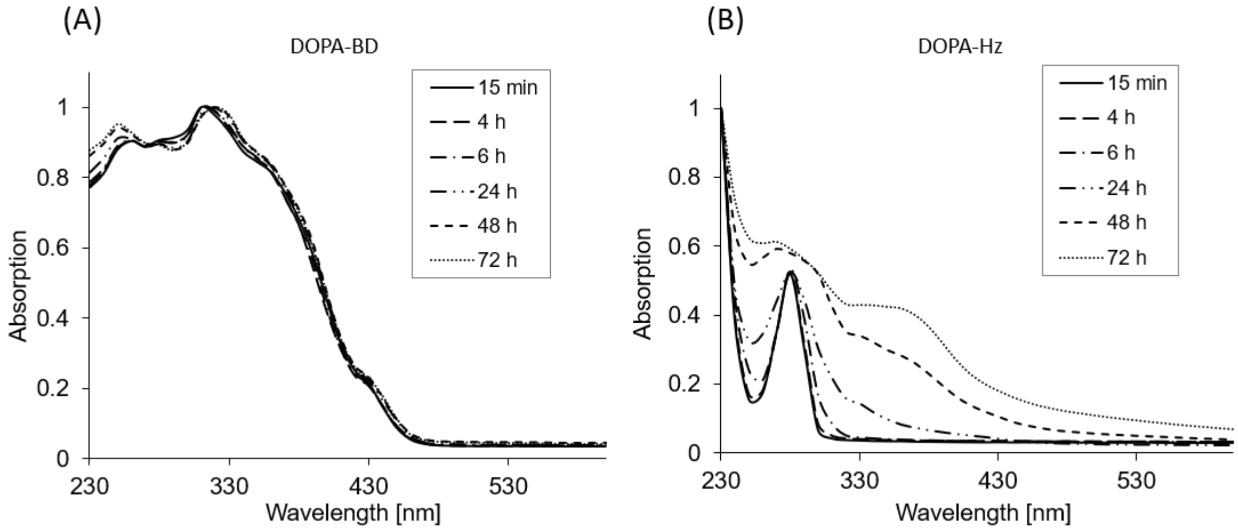

Figure S9. UV-vis absorption spectra of (A) DOPA-BD and (B) DOPA-Hz measured in RPMI medium at pH 7.4.

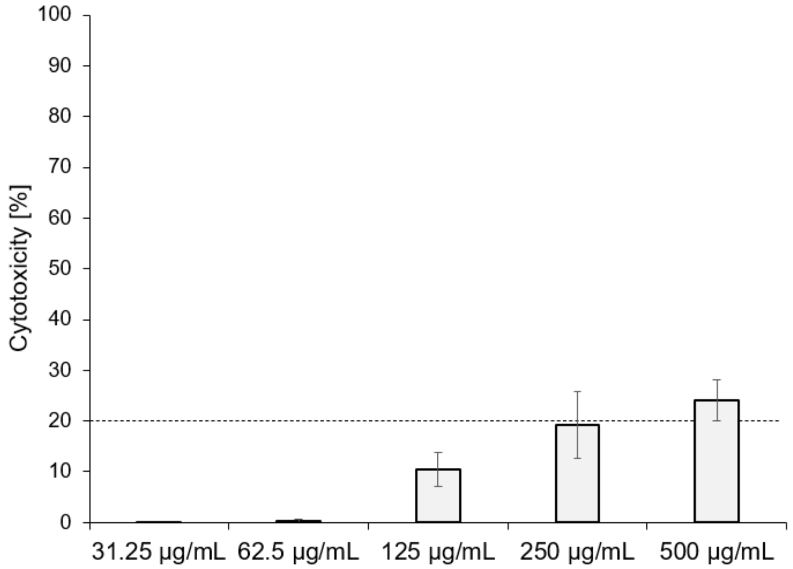

Figure S10. Cytotoxicity of DOPA-BD evaluated by a LDH release assay on A549 cells after 24 h of treatment across a concentration range from 31.25 to 500 µg/mL. The dashed line at 20% indicates the threshold for non-cytotoxic concentrations.[2] Values below this line are considered biocompatible under the applied conditions ($n = 3$, mean ± SD).

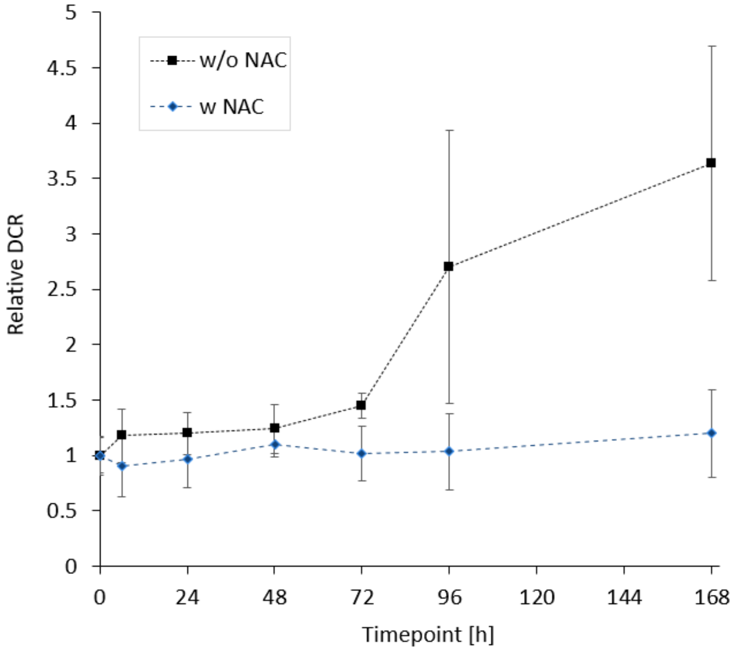

Figure S11. Time-dependent change in relative derived mean count rate (DCR) via DLS analysis of DOPA-BD (black) and DOPA-BD supplemented with 0.5 molar ratio NAC (blue) over a time of 168 h. Data are presented as mean ± SD ($n = 3$).

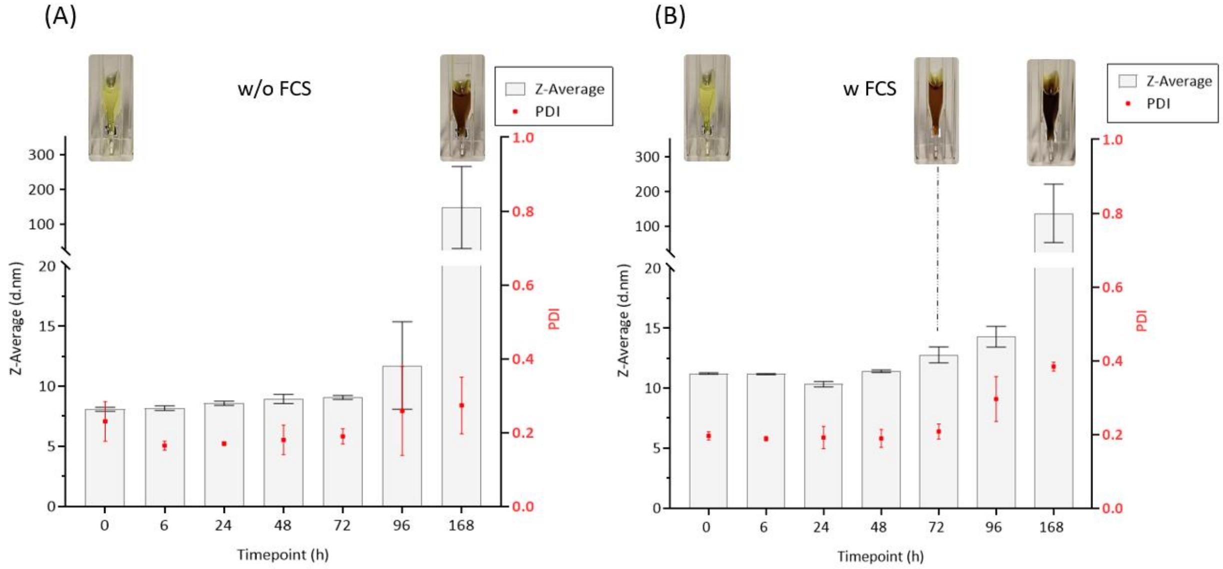

Figure S12. Time-dependent DLS stability analysis of DOPA-BD (512 µg/mL or 0.76 mM) in RPMI medium at different time points until 168 h (7 days). (A) DOPA-BD in RPMI medium without FCS supplementation. (B) DOPA-BD in RPMI medium supplemented with 10% FCS. Data represented as mean ± SD ($n = 3$). It is noted that DLS measurements in FCS-containing

media may be influenced by serum proteins, which can affect the accuracy of absolute D_H-values.

References

(1) Aktaş, N.; Şahiner, N.; Kantoğlu, Ö.; Salih, B.; Tanyolaç, A. Biosynthesis and Characterization of Laccase Catalyzed Poly(Catechol). *J. Polym. Environ.* **2003**, *11* (3), 123–128. DOI: 10.1023/A:1024639231900.

(2) López-García, J.; Lehocký, M.; Humpolíček, P.; Sáha, P. HaCaT Keratinocytes Response on Antimicrobial Atelocollagen Substrates: Extent of Cytotoxicity, Cell Viability and Proliferation. *J. Funct. Biomater.* **2014**, *5* (2), 43–57. DOI: 10.3390/jfb5020043.
